# Sitting Time Reduction and Blood Pressure in Older Adults: A Randomized Clinical Trial

**DOI:** 10.1001/jamanetworkopen.2024.3234

**Published:** 2024-03-27

**Authors:** Dori E. Rosenberg, Weiwei Zhu, Mikael Anne Greenwood-Hickman, Andrea J. Cook, Stefani Florez Acevedo, Jennifer B. McClure, David E. Arterburn, Julie Cooper, Neville Owen, David Dunstan, Stephen R. Perry, Laura Yarborough, Kayne D. Mettert, Beverly B. Green

**Affiliations:** 1 Kaiser Permanente Washington Health Research Institute, Seattle; 2 Kaiser Permanente Bernard J. Tyson School of Medicine, Pasadena, California; 3 Department of Health Services, School of Public Health, University of Washington, Seattle; 4 Baker Heart and Diabetes Institute, Melbourne, Victoria, Australia; 5 Institute for Physical Activity and Nutrition, Deakin University, Burwood, Victoria, Australia

## Abstract

**Importance:**

Practical health promotion strategies for improving cardiometabolic health in older adults are needed.

**Objective:**

To examine the efficacy of a sedentary behavior reduction intervention for reducing sitting time and improving blood pressure in older adults.

**Design, Setting, and Participants:**

This parallel-group randomized clinical trial was conducted in adults aged 60 to 89 years with high sitting time and body mass index of 30 to 50 from January 1, 2019, to November 31, 2022, at a health care system in Washington State.

**Intervention:**

Participants were randomized 1:1 to the sitting reduction intervention or a healthy living attention control condition for 6 months. Intervention participants received 10 health coaching contacts, sitting reduction goals, and a standing desk and fitness tracker to prompt sitting breaks. The attention control group received 10 health coaching contacts to set general healthy living goals, excluding physical activity or sedentary behavior.

**Main Outcomes and Measures:**

The primary outcome, measured at baseline, 3 months, and 6 months, was sitting time assessed using accelerometers worn for 7 days at each time point. Coprimary outcomes were systolic and diastolic blood pressure measured at baseline and 6 months.

**Results:**

A total of 283 participants (140 intervention and 143 control) were randomized (baseline mean [SD] age, 68.8 [6.2] years; 186 [65.7%] female; mean [SD] body mass index, 34.9 [4.7]). At baseline, 147 (51.9%) had a hypertension diagnosis and 97 (69.3%) took at least 1 antihypertensive medication. Sitting time was reduced, favoring the intervention arm, with a difference in the mean change of −31.44 min/d at 3 months (95% CI, −48.69 to −14.19 min/d; *P* < .001) and −31.85 min/d at 6 months (95% CI, −52.91 to −10.79 min/d; *P* = .003). Systolic blood pressure change was lower by 3.48 mm Hg, favoring the intervention arm at 6 months (95% CI, −6.68 to −0.28 mm Hg; *P* = .03). There were 6 serious adverse events in each arm and none were study related.

**Conclusions and Relevance:**

In this study of a 6-month sitting reduction intervention, older adults in the intervention reduced sedentary time by more than 30 min/d and reduced systolic blood pressure. Sitting reduction could be a promising approach to improve health in older adults.

**Trial Registration:**

ClinicalTrials.gov Identifier: NCT03739762

## Key Points

**Question:**

Is sitting time reduction an effective strategy for improving blood pressure?

**Findings:**

In this randomized clinical trial of 283 older adults, there were greater reductions in sitting time and systolic blood pressure during 6 months in the intervention arm compared with the control condition.

**Meaning:**

Reducing sitting time could be a practical strategy for promoting cardiometabolic health in older populations.

## Teaser

This randomized clinical trial of older adults in Washington state assesses whether a sedentary behavior reduction intervention can reduce sitting time and improve blood pressure.

## Introduction

Moderate to vigorous activity can benefit the physical, cognitive, emotional, and functional health of older adults.^1^ However, their levels of meeting physical activity guidelines are low,^2^ with older adults typically sitting for 65% to 80% of waking hours.^3^ Strong evidence from epidemiologic studies associates sedentary behavior with adverse health impacts, including type 2 diabetes, cardiovascular disease, poor physical function, and mortality.^4,5,6,7^ Reducing or breaking up sitting time leads to improved blood pressure (BP) in short-term experimental studies, particularly for those with hypertension.^8,9,10,11^ Given that hypertension prevalence is more than 74% in adults older than 60 years,^12^ finding modifiable factors to improve control of this cardiovascular disease risk factor is crucial.

Sitting reduction interventions with older adults are feasible and can lower sedentary time by approximately 45 to 50 min/d.^13,14,15^ Informed by our preliminary research,^16,17^ the Healthy Aging Resources to Thrive (HART) study was a randomized clinical trial to examine the effectiveness of sitting reduction for improving BP. We hypothesized that participants in the sitting reduction intervention (called I-STAND) would have greater reductions in sitting time and BP (coprimary outcomes) and weight, body mass index (BMI), and waist circumference (secondary outcomes) than those in an attention control group.

## Methods

### Trial Design

We conducted a parallel-group randomized clinical trial with equal randomization (1:1). Recruitment began January 1, 2019, and ended November 31, 2022. Of the 283 randomized patients, 3 withdrew from the intervention and 7 from the control. The main reasons for withdrawal were loss of interest, lack of time, health, or unknown reason (Figure). We designed our study to be geographically inclusive by conducting it primarily remotely and statewide during the COVID-19 pandemic. We oversampled patients of minority racial and ethnic groups to achieve a diverse sample. The intervention was based on 2 pilot studies^16,17^ that included qualitative research to obtain feedback and input for the current design. The study was approved by the Kaiser Permanente Washington (KPWA) Institutional Review Board; the trial protocol is provided in Supplement 1. Before the pandemic, written informed consent was obtained; during the pandemic, digital or written consent was obtained. Study details and adaptations due to COVID-19 have been published.^18^ This study followed the Consolidated Standards of Reporting Trials (CONSORT) reporting guideline.^19^

**Figure.**
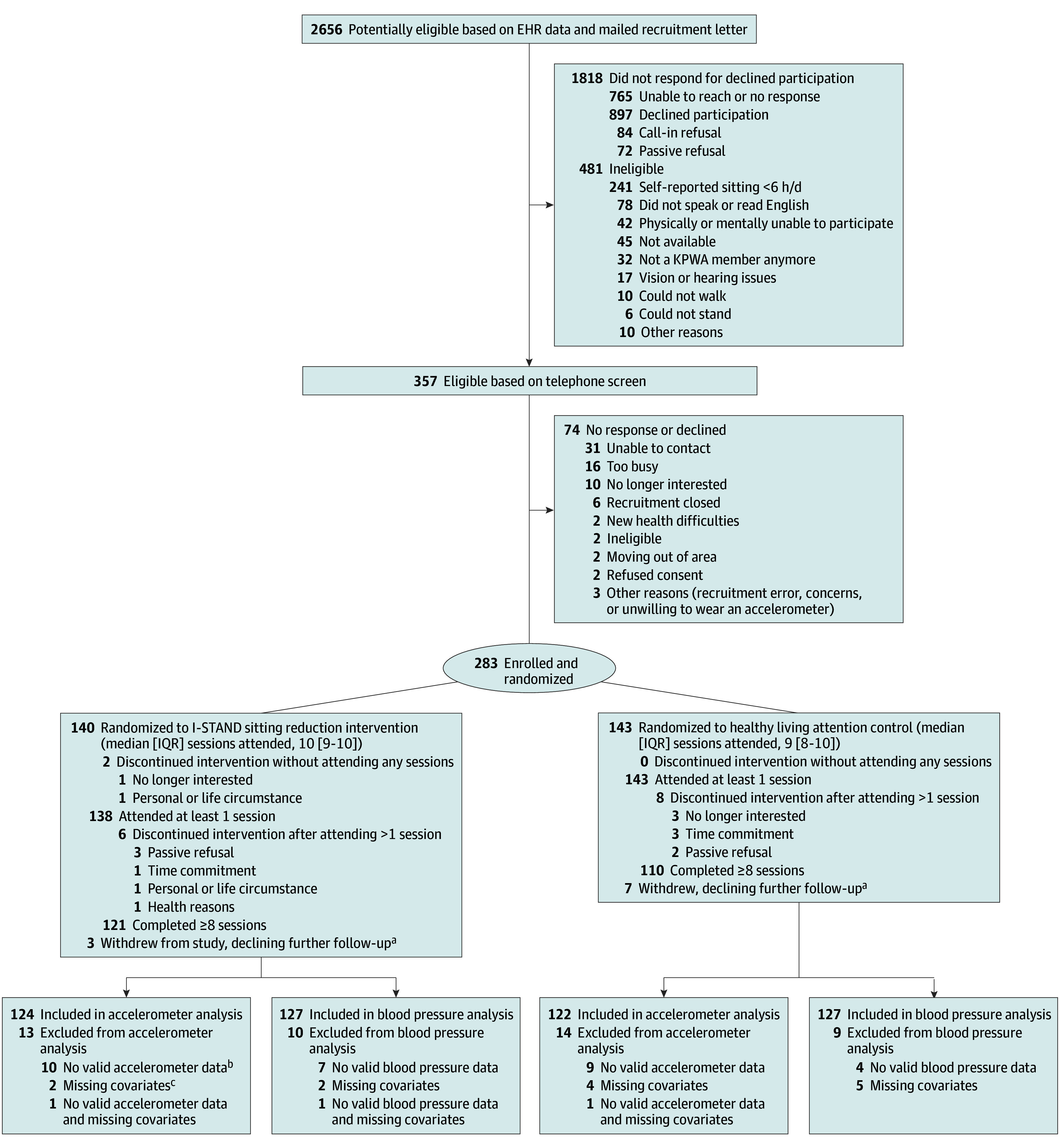
CONSORT Flow Diagram for the Healthy Aging Resources to Thrive (HART) Trial EHR indicates electronic health record; KPWA, Kaiser Permanente Washington. ^a^Participants withdrawing from the overall study is a subset of those discontinuing the intervention (after 0 or ≥1 sessions). Reasons for study withdrawal by arm are as follows: I-STAND: no longer interested (n = 1), health reasons (n = 1), and personal or life circumstance (n = 1); healthy living: no longer interested (n = 3), time commitment (n = 3), and passive refusal (n = 1). ^b^Baseline: outcome is missing or outcome at both month 3 and month 6 are missing. ^c^Baseline covariates included in the regression model are missing.

### Participants and Recruitment

Potential participants were randomly selected from KPWA membership panels, with oversampling of ethnic and racial groups other than non-Hispanic White to achieve inclusivity and sample diversity. Identified individuals were mailed an invitation and pamphlet that described the study and were called approximately 7 days after to assess eligibility and interest.

Participants receiving care at KPWA clinics in King County, Washington (before the pandemic) and statewide (during the pandemic) with electronic health record (EHR) data indicating ages of 60 to 89 years, BMI of 30 to 50 (calculated as weight in kilograms divided by height in meters squared), and continuous enrollment in the prior 12 months were eligible. Exclusions included individuals with *International Statistical Classification of Diseases and Related Health Problems, Tenth Revision* (*ICD-10*) codes in the prior 24 months indicating long-term, palliative, or hospice care; cancer diagnosis; diagnosis of deafness or significant hearing loss; dementia or other degenerative disorder; or a serious mental health disorder. Potentially eligible participants were screened by telephone for additional eligibility criteria, including 6 hours or more of self-reported daily sitting time; not enrolled in other studies at the KPWA; willing to complete study procedures; fluent in English; and able to read study materials, talk on the telephone, stand from a seated position without assistance, and walk 1 block.

### I-STAND Intervention

I-STAND was based on social cognitive theory,^20^ habit formation,^21,22^ and motivational interviewing.^23,24^ Briefly, I-STAND participants received 10 brief health coaching contacts (2 in person and 8 by telephone before the pandemic and all by telephone during pandemic) during 6 months and a study workbook, generic wrist-worn fitness band (exact brand depended on supply chain availability), tabletop standing desk, and feedback report after wearing the accelerometers (activPAL, PAL Technologies Ltd) at baseline and 3 months. Participants set individualized goals on reducing sitting time by standing more and taking frequent sitting breaks. Participants developed personalized reminder systems consisting of inner reminders (eg, noticing muscle stiffness), external reminders (eg, fitness band prompts), and habit reminders (eg, adding standing to habitual activities, such as reading the newspaper) to break automatic and habitual sitting patterns.

### Healthy Living Attention—Control Condition

Control participants received 10 health coaching contacts during 6 months and a workbook. At each contact, participants chose 1 topic to review from a list of relevant healthy living topics (eg, fall prevention, healthy eating, sleep) with their health coach and set topic-related goals.

### Health Coach Training

Health coaches ranged in experience from no health coaching experience to degrees in social work, nursing, or public health. All were trained in motivational interviewing and the content of both interventions by the lead researcher (D.E.R.), a licensed clinical psychologist. Training included 4 hours of didactic training and at least 10 hours of shadowing and practice and ongoing supervision.

### Fidelity and Adherence

Semistructured session guides were used to standardize health coaching in each arm. Approximately 10% of the intervention group and 6% of the attention control group sessions were coded for fidelity by a trained coder using a structured coding protocol and checklist. The checklist included adherence to motivational interviewing principles and intervention content. Coaches received feedback based on the fidelity review. We tracked adherence rates by the number of health coach sessions completed.

### Outcomes

#### Primary Outcomes

The primary outcome was changes in daily sitting time (in minutes) measured by an accelerometer (activPAL) at baseline, 3 months, and 6 months. The coprimary outcome was changes in systolic blood pressure (SBP) and diastolic blood pressure (DBP) measured at baseline and 6 months (3-month measures were available on a subsample captured during the COVID-19 pandemic). Participants wore an accelerometer validated for sitting^25,26,27^ on their thigh for 7 days. Participants kept sleep logs, which were used as an indicator of waking wear time. Sleep was removed from the accelerometer analytic data. Accelerometer events files were processed through a program in R software (R Project for Statistical Computing) to summarize mean daily waking sitting time (in minutes). Accelerometer data were valid if waking wear time was greater than 10 hours per day with a minimum of 4 valid days of data for each assessment period.^24,28,29^ To account for variations in waking wear time (which can greatly influence sitting outcomes), sitting time was standardized by calculating the mean sitting time during a 15-hour wear period averaged over days of valid wear.

Blood pressure was assessed on participants’ nondominant arm using an Omron BP device (HEM-907XL^30,31^ before the pandemic and BP5100 and HEM-7311 or equivalent BP7100^32,33^ during the pandemic) after 5 minutes of rest.^18,34^ Of 3 measures, the mean of the last 2 was used for analysis. Research assessors received training in BP measurement according to American Heart Association practice guidelines.^35,36^ After the pandemic onset, research assessors guided participants in taking their own BP by telephone.

#### Secondary Outcomes

Stadiometers (model 213, Seca) were used to measure height before the pandemic and self-reported during the pandemic. Weight was measured with a calibrated digital scale (Seca) and a digital scale (Etekcity) before and during the pandemic, respectively. Waist circumference was measured at the superior border of the iliac crest, in person before the pandemic or with coaching by telephone during the pandemic.

#### Exploratory Outcomes

Changes in accelerometer standing and stepping time (in minutes per day), daily step count, number of sitting bouts of 30 minutes or greater, and mean sitting bout duration were derived using the process described for sitting time. Time (in seconds) to complete 5 chair stands from the Short Physical Performance Battery^37,38,39^ was conducted (in person before the pandemic and by telephone during the pandemic). Change in hypertension medication classes at 6 months from baseline was defined as decreasing, staying the same, or increasing from EHR data.

#### Covariates

Prespecified covariates included age, gender, race, and ethnicity (measured from self-report or EHRs where missing [18 participants]), county of residence, physical function (self-reported ability to walk at a normal pace), retirement status, presence of diabetes and hypertension diagnoses in the EHR, and number of hypertension medication classes. Race and ethnicity were included as social constructs to represent, although in a limited manner, some exposure to systemic racism and health care inequities.^40^ Due to imbalances across groups, self-reported arthritis was included. Body mass index was further included.

### Randomization and Masking

After baseline primary outcomes were collected, participants were randomized by their health coach using a computer-generated concealed randomization scheme, created by the lead statistician (A.J.C.), in a 1:1 allocation to I-STAND or the healthy living control. Randomization was stratified by baseline self-reported sitting time (<8.5 vs ≥8.5 h/d), SBP higher than 130 mm Hg (yes vs no), diabetes diagnosis (yes vs no), and BMI greater than 35 (yes vs no). Staff who collected measures and study statisticians were masked to participants’ treatment arm. Participants and health coaches were aware of treatment assignment. Before the pandemic, randomization and intervention start were concurrent, whereas during the pandemic there was an approximately 2-week lag to allow for mailing of materials.

### Adverse Events

Participants reported changes in their health at each coaching session. A physician not involved in the conduct of the study determined whether events were study related. Expected adverse events included mild muscle soreness, fatigue, stiffness, skin irritation from the accelerometer, and increased risk of falls.

### Sample Size

The sample size of 284 ensured 90% power to detect a 41-minute difference in sitting time between I-STAND and the attention control from baseline to 6 months, assuming an SD of 97 min/d for change in sitting time (based on our pilot data), and 15% loss to follow-up. A sample size of 241 provided 90% power to detect a mean (SD) difference of 6.2 (14.7) mm Hg change in SBP and 4.0 (9.5) mm Hg change in DBP. We a priori defined sitting time and BP as coprimary outcomes, which requires reporting of all coprimary outcomes in describing the results of the study (eg, reducing sitting time without improving a BP outcome would not be an overall positive study); in addition, these outcomes could have independent mechanisms from the I-STAND intervention (BP could be changed not only through reduced sitting), so we did not adjust for multiple comparisons.^41,42^ Sample and power calculations were performed using R software, version 3.5 via simulation, assuming specified SDs and differences between means.^43^

### Statistical Analysis

All analyses were preplanned,^18^ used intention-to-treat approaches, and were in accordance with recommendations.^44^ Wald statistics with 2-sided 95% CIs and *P* values were used, and statistical significance was specified at an α = .05. All analyses were conducted in Stata, version 17 (StataCorp LLC). Descriptive statistics of baseline demographics, health, and accelerometer measures by intervention group (I-STAND vs control) were conducted.

#### Primary Outcome Analyses

For each coprimary outcome (accelerometer sitting time, SBP, and DBP), both 3-month and 6-month (primary) time points were included in a single model and fit a linear regression model using generalized estimating equations to estimate the difference between the adjusted mean changes from baseline between participants randomized to I-STAND vs control.^45^ Each model included all participants with a given outcome at either 3 or 6 months. Regression models included intervention assignment, time point indicator, and interaction between intervention assignment and time point indicator as well as baseline outcome, covariates, and interaction between baseline outcome and COVID-19 indicator (defined as randomization before vs during the pandemic). Secondary analysis was conducted by further including a COVID-19 interaction term to assess for differential effects of the intervention before and during the pandemic.

Finally, a COVID-19 sensitivity analysis was conducted, fitting separate models among those with all measurements taken before the pandemic and all measurements taken during the pandemic. This analysis assessed the potential differential measurement error in our outcomes between baseline measures collected in clinic before the pandemic and at home during pandemic. Both the COVID-19–related secondary analysis and the sensitivity analyses have limited power due to smaller sample sizes. Nonetheless, all were preplanned to assess the effect of the pandemic on our trial results.

#### Missing Data

Preplanned adjustment for baseline characteristics associated with outcome, loss to follow-up, or observed differential by randomization group was applied to account for potential bias due to missing data. As prespecified,^18^ if the amount of missing data was higher than 15%, we planned to use nonignorable missing data imputation methods. This threshold was not met for any primary outcomes and analyses were not conducted.

#### Secondary Outcomes, Exploratory Outcomes, and Moderators

Analyses for secondary and exploratory outcomes followed the primary outcome analyses except for medication changes for which, due to small numbers, an unadjusted χ^2^ test was used. Exploratory moderator analyses for primary outcomes were conducted at 6 months using the primary outcome analysis framework (excluding the 3-month outcome data) and including interactions with each baseline moderator and intervention.

## Results

### Patient Characteristics

At baseline, the 283 participants had a mean (SD) age of 68.8 (6.2 years); 186 (65.7%) were female, 96 (33.9%) were male, and 1 (0.4%) was nonbinary; and 1 (0.4%) was Arab American or Lebanese, 9 (3.2%) were Asian, 41 (14.5%) Black, 6 (2.1%) Hawaiian or Pacific Islander, 16 (5.7%) Hispanic or Latino, 3 (1.1%) Native American or American Indian, 195 (68.9%) White, and 12 (4.2%) multiracial. Participants had a mean (SD) BMI of 34.9 (4.7). A total of 147 (51.9%) had a hypertension diagnosis, and 97 (69.3%) took at least 1 antihypertensive medication (Table 1). Accelerometer awake time was approximately 15.5 hours per day across 7 days of wear for both groups.

**Table 1.** Demographics, Health Characteristics, and Study Outcomes at Baseline for the Total Study Population and by Intervention Group^a^

Baseline characteristic	Total (N = 283)	Attention control (n = 143)	Intervention (n = 140)
Age, mean (SD), y^b^	68.8 (6.2)	69.0 (6.5)	68.6 (6.0)
Age, group, y			
≤74	228 (80.6)	115 (80.4)	113 (80.7)
>74,	55 (19.4)	28 (19.6)	27 (19.3)
Gender^b^^,^^c^			
Female	186 (65.7)	99 (69.2)	87 (62.1)
Male	96 (33.9)	44 (30.8)	52 (37.1)
Nonbinary^d^	1 (0.4)	0	1 (0.7)
Race and ethnicity^b^^,^^c^^,^^e^			
Asian	9 (3.2)	5 (3.5)	4 (2.9)
Black	41 (14.5)	16 (11.2)	25 (17.9)
Hawaiian or Pacific Islander	6 (2.1)	2 (1.4)	4 (2.9)
Hispanic or Latino	16 (5.7)	10 (7.0)	6 (4.3)
Lebanese	1 (0.4)	0	1 (0.7)
Native American or American Indian	3 (1.1)	2 (1.4)	1 (0.7)
White	195 (68.9)	102 (71.3)	93 (66.4)
Multiracial	12 (4.2)	6 (4.2)	6 (4.3)
Retired^c^	157 (55.5)	79 (55.2)	78 (55.7)
Marital status^c^			
Married or living as married	175 (61.8)	90 (62.9)	85 (60.7)
Single never married	23 (8.1)	14 (9.8)	9 (6.4)
Widowed, separated, or divorced	76 (26.9)	34 (23.8)	42 (30.0)
Educational level^c^			
College or higher	149 (52.7)	79 (55.2)	70 (50.0)
Some college or vocational school	113 (39.9)	51 (35.7)	62 (44.3)
High school or GED	10 (3.5)	5 (3.5)	5 (3.6)
Some high school or less	2 (0.7)	1 (0.7)	1 (0.7)
King County residence^b^	155 (54.8)	74 (51.7)	81 (57.9)
Randomized during the COVID-19 pandemic	183 (64.7)	96 (67.1)	87 (62.1)
Self-rated health^c^			
Poor, fair, or good	194 (68.6)	97 (67.8)	97 (69.3)
Very good or excellent	80 (28.3)	38 (26.6)	42 (30.0)
Fall in last 12 mo^c^	102 (36.0)	53 (37.1)	49 (35.0)
Able to walk at normal pace^c^	222 (78.4)	111 (77.6)	111 (79.3)
Depression symptoms^c^	13 (4.6)	5 (3.5)	8 (5.7)
Current smoker^c^	2 (0.7)	0	2 (1.4)
Elixhauser Comorbidity Index (weighted with cancer), mean (SD)^b^	1.7 (1.7)	1.8 (1.9)	1.7 (1.4)
Self-reported arthritis^c^	122 (43.1)	52 (36.4)	70 (50.0)
Diabetes^b^	80 (28.3)	41 (28.7)	39 (27.9)
Hypertension *ICD-10* diagnosis code in prior year^b^	147 (51.9)	73 (51.0)	74 (52.9)
Hypertension medication classes, No.^b^			
0	87 (30.7)	40 (28.0)	47 (33.6)
1	84 (29.7)	46 (32.2)	38 (27.1)
2	64 (22.6)	31 (21.7)	33 (23.6)
≥3	48 (17.0)	26 (18.2)	22 (15.7)
SBP, mean (SD), mm Hg^f^	136.1 (18.5)	136.7 (18.3)	135.4 (18.8)
DBP, mean (SD), mm Hg^f^	79.7 (10.7)	79.8 (10.2)	79.6 (11.1)
Weight, mean (SD), kg^f^	98.30 (15.69)	97.89 (15.88)	98.70 (15.60)
BMI, mean (SD) ^f^	34.9 (4.7)	35.2 (4.8)	34.7 (4.6)
Waist circumference, mean (SD), cm^f^	113.54 (12.70)	114.05 (13.21)	112.78 (11.94)
Time to do 5 chair stands, mean (SD), s^f^	15.1 (5.9)	15.0 (5.7)	15.3 (6.1)
Daily accelerometer sitting time, mean (SD), min/d^f^	652.4 (116.5)	654.5 (115.5)	650.4 (117.9)
Daily accelerometer standing time, mean (SD), min/d^f^	206.8 (94.1)	206.1 (95.0)	207.4 (93.4)
Daily accelerometer stepping time, mean (SD), min/d^f^	71.4 (31.2)	70.1 (31.3)	72.7 (31.2)
Daily total accelerometer steps per d, mean (SD)^f^	5377 (2590.5)	5263 (2537.7)	5492 (2646.9)
Daily total accelerometer steps per day ≥5000^f^	135 (47.7)	62 (43.4)	73 (52.1)
Daily accelerometer sitting bout duration, mean (SD), min/d^f^	17.8 (7.6)	18.6 (8.5)	17.1 (6.5)
No. of accelerometer sitting bouts lasting ≥30 min, mean (SD)^f^	6.5 (1.7)	6.5 (1.7)	6.5 (1.8)

Abbreviations: BMI, body mass index (calculated as weight in kilograms divided by height in meters squared); DBP, diastolic blood pressure; GED, General Educational Development; *ICD-10, International Statistical Classification of Diseases and Related Health Problems, Tenth Revision*; SBP, systolic blood pressure.

^a^ Data are presented as number (percentage) of participants unless otherwise indicated. Data were missing for the following: retirement, 8; marital status, 9; educational level, 9; self-rated health, 9; fall in last 12 months, 7; able to walk at a normal pace, 5; depression symptoms, 19; current smoker, 11; Elixhauser Comorbidity Index, 3; SBP, 1; DBP, 1; waist circumference, 6; time to chair stand, 8; and all accelerometer variables, 6.

^b^ Data are from the electronic health record.

^c^ Self-reported data are from the online survey.

^d^ In analyses, 1 participant who identified as nonbinary in study questionnaires was grouped into the women category based on their sex at birth from electronic medical records.

^e^ All races are non-Hispanic.

^f^ Data are from this study.

Overall, 121 participants (86.4%) in the intervention group and 110 (76.9%) in the control group completed 8 or more sessions (Figure). Fidelity was high in both arms; sedentary behavior topics were discussed in 3 of 85 coded control sessions (3.5%) (eTable 1 in Supplement 2).

In the intervention group (n = 140), 124 (88.6%) had valid accelerometer data at 3 or 6 months and were included in accelerometer analyses, whereas 127 (90.7%) were included in BP analyses. In the control group (n = 143), 122 (85.3%) had valid data for accelerometer analyses and 127 (88.8%) for BP analyses at 3 or 6 months.

### Primary Analysis

Mean change in sitting time decreased significantly more in the intervention arm compared with the control group by 31.85 min/d (95% CI, −52.91 to −10.79 min/d; *P* = .003) at 6 months (the eFigure in Supplement 2 provides a waterfall plot of individual changes in sitting time). Furthermore, mean change in SBP decreased significantly more, favoring the intervention arm at 6 months (−3.48 mm Hg; 95% CI, −6.68 to −0.28 mm Hg; *P* = .03). Diastolic BP was not significantly different between groups (0.27 mm Hg; 95% CI, −1.63 to 2.16 mm Hg; *P* = .78) (Table 2).

**Table 2.** Coprimary Outcome and Secondary Outcome Results by Intervention Group

Measure	Adjusted mean change (95% CI)^a^		Difference in adjusted mean changes (95% CI)^a^	*P* value
	Attention control	I-STAND intervention		
**Primary outcomes**				
Sitting time (n = 246)				
3 mo	−6.94 (−17.36 to 3.49)	−38.38 (−51.80 to −24.96)	−31.44 (−48.69 to −14.19)	<.001
6 mo^b^	−8.51 (−23.97 to 6.95)	−40.36 (−54.86 to −25.85)	−31.85 (−52.91 to −10.79)	.003
SBP at 6 mo (n = 254) ^b^	−3.19 (−5.44 to −0.94)	−6.67 (−8.82 to −4.52)	−3.48 (−6.68 to −0.28)	.03
DBP 6 mo (n = 254)^b^	−1.58 (−2.93 to −0.23)	−1.32 (−2.66 to 0.02)	0.27 (−1.63 to 2.16)	.78
**Secondary outcomes**				
Weight at 6 mo (n = 210)	−3.45 (−4.95 to −1.94)	−3.56 (−5.50 to −1.63)	−0.12 (−2.61 to 2.38)	.93
BMI at 6 mo (n = 210)	−0.54 (−0.81 to −0.26)	−0.51 (−0.85 to −0.17)	0.02 (−0.42 to 0.47)	.92
Waist circumference at 6 mo (n = 248)	−0.64 (−1.09 to −0.19)	−1.00 (−1.55 to −0.45)	−0.36 (−1.09 to 0.36)	.33

Abbreviations: BMI, body mass index (calculated as weight in kilograms divided by height in meters squared); DBP, diastolic blood pressure; SBP, systolic blood pressure.

^a^ For adjusted mean change and difference, we fit a linear regression model with generalized estimating equations on the outcome change from baseline, including the time points 3 and 6 months in the same model with indicators for 6-month time point, I-STAND intervention, and interactions among these indicators, adjusting for baseline outcome, post–COVID-19 randomization indicator, baseline outcome and post–COVID-19 interaction, county, age, gender, race, BMI, arthritis, physical function, diabetes, hypertension, retirement status, and number of hypertensive medication classes. To obtain the adjustment mean change, we assumed the mean baseline covariate response for all randomized participants. For SBP and DBP, we give only 6-month outcomes because 3-month outcome data were collected only on participants enrolled during the COVID-19 pandemic.

^b^ Six months is the primary time point of the trial.

At 6 months, mean change in sitting reduction was larger for those randomized during the pandemic (−40.25 min; 95% CI, −65.39 to −15.11 min; *P* = .04) (eTable 2 in Supplement 2). The decrease in SBP was similar for those randomized before (−3.75 mm Hg; 95% CI, −7.27 to −0.23 mm Hg; *P* = .27) or during (−3.41; 95% CI, −9.47 to 2.66 mm Hg; *P* = .04) the pandemic.

### Sensitivity Analyses

There were statistically significant reductions in sitting time that favored the intervention group at 3 and 6 months regardless of whether measurements were taken before or during the pandemic (eTable 3 in Supplement 2). Differences in SBP were not statistically significant, although there was an observed effect similar to the primary results during the pandemic (difference in adjusted mean change, −3.18 mm Hg; 95% CI, −6.75 to 0.39 mm Hg; *P* = .08).

### Secondary and Exploratory Outcomes

No statistically significant between-group differences were observed for weight, BMI, or waist circumference (Table 2). Mean change in standing time increased, whereas mean sitting bout duration and prolonged sitting bouts decreased at 6 months, favoring the intervention group; all findings were statistically significant (Table 3). In the control group, 5 people (3.2%) decreased and 6 (4.2%) increased hypertension medication classes, whereas in the intervention group, 10 people (7.1%) decreased and 8 (5.7%) increased the number of hypertension medication classes, but these differences were not statistically significant.

**Table 3.** Exploratory Outcomes by Intervention Group at 6 Months

Measure	Adjusted mean change (95% CI)^a^		Difference in adjusted mean changes (95% CI)^a^	*P* value
	Attention control	I-STAND intervention		
Standing time, min/d				
3 mo	4.46 (−5.00 to 13.91)	31.58 (19.97 to 43.20)	27.13 (12.00 to 42.26)	<.001
6 mo^b^	4.81 (−8.67 to 18.29)	32.48 (19.64 to 45.33)	27.67 (9.30 to 46.04)	.003
Stepping time, min/d				
3 mo	2.46 (−0.88 to 5.80)	6.81 (3.26 to 10.36)	4.35 (−0.60 to 9.30)	.09
6 mo^b^	3.77 (−0.40 to 7.94)	7.81 (4.12 to 11.50)	4.04 (−1.59 to 9.67)	.16
Mean daily step count				
3 mo	212.07 (−126.05 to 550.19)	568.15 (249.63 to 886.68)	356.08 (−110.38 to 822.54)	.14
6 mo^b^	352.42 (−36.62 to 741.46)	510.14 (175.52 to 844.75)	157.72 (−356.73 to 672.16)	.55
Mean sitting bout duration, min/d				
3 mo	−0.43 (−1.25 to 0.39)	−1.35 (−2.37 to −0.33)	−0.92 (−2.33 to 0.49)	.20
6 mo^b^	−0.04 (−0.95 to 0.87)	−1.85 (−2.73 to −0.98)	−1.81 (−3.11 to −0.52)	.006
Prolonged sitting bouts, No.				
3 mo	−0.18 (−0.40 to 0.04)	−0.72 (−0.98 to −0.45)	−0.54 (−0.89 to −0.18)	.003
6 mo^b^	−0.13 (−0.40 to 0.14)	−0.70 (−0.98 to −0.42)	−0.57 (−0.96 to −0.18)	.004
Chair stands, s				
3 mo	−0.53 (−1.41 to 0.35)	−1.29 (−2.09 to −0.49)	−0.76 (−1.95 to 0.43)	.21
6 mo^b^	−1.20 (−2.06 to −0.34)	−1.15 (−2.14 to −0.16)	0.05 (−1.29 to 1.39)	.95

^a^ For adjusted mean change and difference, we fit a linear regression model with generalized estimating equations on the outcome change from baseline, including 3- and 6-month time points in the same model with indicators for the 6-month time point, I-STAND intervention, and interactions among these indicators, adjusting for baseline outcome, post–COVID-19 randomization indicator, baseline outcome and post–COVID-19 interaction, county, age, gender, race, body mass index, arthritis, physical function, diabetes, hypertension, retirement status, and number of hypertensive medication classes. To obtain the adjusted mean change, we assumed the mean baseline covariate response for all randomized participants.

^b^ Six months is the primary time point of the trial.

### Moderators

No moderators were statistically significantly different between groups with 2 exceptions (eTable 4 in Supplement 2). Women had statistically significant decreases in DBP compared with men, but the effect was small; similarly, participants residing in more suburban and rural areas had small decreases in DBP, favoring the intervention group.

### Harms

The intervention and control groups reported 87 and 57 adverse events, respectively. The intervention group had more musculoskeletal events (35 vs 19 in the control group) and more skin disorders (9 vs 1 in the control group). Six serious adverse events were reported in the intervention arm (2 cancer diagnoses, 1 fall, 1 emergency department admission, and 2 hospitalizations) and 6 in the control arm (1 cancer diagnosis, 1 fall, and 4 hospitalizations). No serious adverse events were study related.

## Discussion

The sitting reduction intervention was effective at reducing sitting time by more than a half-hour per day during 6 months. It also increased standing time and reduced prolonged sitting periods. There were meaningful improvements in SBP of nearly 3.5 mm Hg that were commensurate to the effects of aerobic physical activity interventions.^46^ For comparison, systematic reviews indicate that aerobic physical activity reduces SBP by 4 mm Hg,^46^ the Dietary Approach to Stop Hypertension (DASH) diet by 5.2 mm Hg,^47^ and weight loss by 3 mm Hg.^48^ Sitting reduction through standing more and taking more frequent breaks from sitting may be a novel lifestyle strategy for improving BP and easier for older individuals with chronic conditions to incorporate into their daily life.^49,50^ Potential physiologic reasons for SBP reductions could include more frequent interruptions to the bent artery position, which could improve blood flow and vascular shear stress.^51^

Our finding that sitting was reduced by approximately 31 minutes per day is slightly lower than others have found^52^ and did not meet the study goal of a reduction of 2 hours per day. A Cochrane review of interventions to reduce sitting in older adults, including 7 randomized clinical trials, found an overall reduction of approximately 45 minutes per day, favoring intervention groups.^14^ In addition, most of our participants were affected by COVID-19 pandemic restrictions. Furthermore, the amount of sedentary behavior reduction needed to confer health benefits is not known.

Nonetheless, our intervention resulted in significant improvements in BP. This may be because we recruited participants at high risk of hypertension and aimed to reduce sitting time as well as decrease prolonged sitting. Perhaps given a population with higher cardiovascular risk, small changes in sitting patterns were sufficient for improving BP. The promising finding of no significant effect modifiers indicated that people with lower physical function or physical activity or with chronic conditions, such as type 2 diabetes, can reduce their sitting time and reap BP benefits. Decreased sitting could serve as a gateway to more physical activity as people gain strength and confidence.

### Strengths and Limitations

Strengths of our study include use of an attention control group, a robust design, sufficient sample size for primary outcome analyses, and a 6-month period to observe health outcomes with high adherence and low dropout rates. The intervention caused no additional harm to participants, which is reassuring given the older age group.

Limitations of our study include the interruption caused by the COVID-19 pandemic, which occurred after 100 of the 283 participants were enrolled. We had to rely on participant-measured outcomes during the pandemic. Compared with the state population, we had fewer participants who identified as Asian or Hispanic or Latino. Furthermore, because intervention participants received accelerometer feedback charts, they may have been more likely to change their behavior while wearing the accelerometer. Our effects on BP might have been greater had we excluded normotensive individuals.

## Conclusions

This randomized clinical trial showed that an intervention to reduce sitting time can be successfully delivered remotely and result in significant reductions in sitting time. These changes led to meaningful reductions in BP. Interventions that result in less sitting and more standing breaks deserve further study because they could lead to improved cardiovascular outcomes.

## Notes

Supplement 1.
Trial Protocol and Statistical Analysis Plan

Supplement 2. **eTable 1.** Adherence to Health Coaching Sessions and Fidelity to the Interventions **eTable 2.** Coprimary Outcome Secondary Analysis at 3 and 6 Months by Intervention Group and Randomization Pre or Post COVID-19 Pandemic **eTable 3.** Coprimary Outcomes Results by Intervention Group at 3 and 6 Months by Whether All Measurements Were Completed Before or After the COVID-19 Pandemic Onset **eTable 4.** Moderator Analyses at 6 Months to Assess Differential Intervention Effects **eFigure.** Waterfall Plot of Changes From Baseline to 6 Months in Mean Daily Minutes of Sitting Time

Supplement 3.
Data Sharing Statement
