# Supplementary material for: Sitting Time Reduction and Blood Pressure in Older Adults: A Randomized Clinical Trial
Source: JAMA Netw Open. 2024 Mar 27;7(3):e243234. doi: 10.1001/jamanetworkopen.2024.3234 (PMC10973891; doi:10.1001/jamanetworkopen.2024.3234)
Supplement: Supplement 1. — Trial Protocol and Statistical Analysis Plan [file jamanetwopen-e243234-s001.pdf]

KAISER PERMANENTE PROTOCOL FORM & STATISTICAL ANALYSIS PLAN (P 44)

Health Aging Resources to Thrive (HART)

NCT03739762

IRB Approval Date: April 14, 2022

Protocol Title
Randomized Trial to Reduce Sitting Time and Improve Cardiometabolic Health in Obese Older Adults

Principal Investigator
Dori Rosenberg, PhD, MPH

Version Date
Approved April 14, 2022

Form Author
Julie Cooper

1. Objectives

Describe in plain language the purpose, specific aims, or objectives and indicate the primary goal(s) of the study (e.g. safety, tolerability, effectiveness, feasibility, pilot study, etc.). State the hypotheses to be tested. State primary and any secondary study endpoints.

Purpose:

Healthy Aging Resources to Thrive (HART) intervention focuses on reducing sitting time by standing more and breaking up prolonged bouts of sitting throughout the day. Because older adults with obesity rarely engage in physical activity, helping them sit less throughout the day could be a meaningful, but untested, health-promoting behavior. We will target a group with high prevalence of cardiometabolic conditions in which we have the opportunity to see the largest improvement in biomarkers related to cardiometabolic risk.[1-3] We will conduct an efficacy randomized controlled trial to determine whether a sitting reduction intervention, improves behavioral and health outcomes to a greater extent than an attention-control group. The primary end point is 6 months. but participants randomized to the i-STAND intervention will be re-randomized at 6 months to either receive no further contact or a booster session intervention. Everyone will be followed until 12 months after baseline.

2022 BUDGET CUT UPDATE: As of March 31, 2022, participants randomized to the i-STAND intervention will no longer be re-randomized at 6 months. We will no longer follow participants to the 12-month timepoint and will end all activities after the 6-month timepoint.

The specific aims of the study are to:

Aim 1: Determine the efficacy of i-STAND to improve primary and secondary outcomes at 6 months.

Our primary outcomes are change from baseline in mean daily sitting time and systolic and diastolic blood pressure. We will compare group means for our primary outcomes between participants randomized to i-STAND versus attention control (“Healthy Lifestyles”) at 6 months. Secondary outcomes include cardiometabolic biomarkers (HbA1c,), weight, height, and physical function.

Aim 2: Determine the maintenance of sitting and blood pressure reductions over 12 months.

We will then assess differences in primary outcomes at 12 months in the two i-STAND groups: with and without the booster intervention.

Hypothesis 2: i-STAND participants randomized to receive booster sessions will have reduced sitting time and improved cardiometabolic risk factors at 12 months compared to i-STAND participants receiving no further intervention and compared to the Healthy Lifestyles/attention control participants.

2022 BUDGET CUT UPDATE: As of March 31, 2022, we are cutting Aim 2 so as to conserve funds to focus on Aims 1 and 3.

Aim 3: Explore moderators (e.g., age, sex, baseline physical activity) and mediators of reductions in sitting time (e.g., psychosocial variables) and cardiometabolic risk factors (e.g., changes in sitting time and/or physical activity).

Additional exploratory analyses: We will explore whether i-STAND improves standing time, breaks from sitting, physical activity (step counts as measured by activPAL), quality of life, physical function, and depressive symptoms. Further, per protocol, analyses amongst those that adhered to the intervention will also be conducted.

2. Background

a. Scientific Background

Provide the scientific or scholarly background for, rationale for, and significance of the research based on the existing literature and how will it add to existing knowledge. A list of references or bibliography must be included as part of this document or uploaded separately.

Currently, about 40% of American adults over 60 are obese, defined as BMI ≥30.[4,5] . Increased physical activity, particularly at moderate-to-vigorous levels, promotes healthy weight and has a plethora of positive effects on cardiometabolic conditions.[6,7] Yet, of all age groups, older adults are the least likely to meet physical activity guidelines (2.4% of adults aged 65 and older by objective measures).[8] Even with public health efforts to promote leisure time activity, rates are unchanged over the past 10 years.[9,10] Obese adults are even less likely than overweight and normal weight adults to be physically active.[11-13] Many older adults face considerable barriers to physical activity including pain, fear, incontinence, fatigue, depression, lack of confidence, cost, safety, weather, and lack of facilities and transportation.[12,14] Many older adults cannot appreciably increase their level of physical activity and reducing sedentary behavior could be a more feasible alternative. A meta-analysis of 41 primarily prospective studies of self-reported sedentary time and health outcomes (including in older adults) found that, after adjusting for physical activity, individuals with higher sedentary time had greater risk of type 2 diabetes, all-cause mortality, incident CVD, and CVD mortality, with hazard ratios from 1.14 (incident CVD) to 1.91 (diabetes).[15] Sitting-reduction interventions in free-living conditions generally focus on younger adults in workplace settings and reduce sitting by an average of 77 minutes/8-hour workday;[55] several have successfully improved waist circumference,[16] blood pressure,[17] and blood lipid profiles.[16,18] Our own pilot work demonstrates that reducing sitting time is feasible and acceptable in older adults with obesity.[19,20]

We now wish to determine, in the first large scale RCT in an older population, whether reductions in sitting are associated with health outcomes at 6 months and 1-year post-baseline.
2022 BUDGET CUT UPDATE: As of March 31, 2022, we are no longer collecting measures at 1 year.

b. Preliminary Data

Describe any relevant preliminary data.

Our main preliminary study was a recently completed pilot RCT in obese adults over age 60 (PI: Rosenberg). We found high levels of acceptability and excellent adherence. Sitting significantly reduced and standing significantly increased for those receiving the intervention.

Table 1. Preliminary sitting reduction studies from the study team

Study & design	Intervention(s)	Results	KPWA IRB Info
**Kerr** [21]<br>Randomized pilot over 3 weeks (N = 30; aged 50-70, Mean BMI = 27, 50% working, 50% retired)	Group 1: Increase standing time using standing desks.<br>Group 2: Increase breaks from sitting using prompting strategies. Both met in-person weekly with health educator.	Group 1: -130 min/day in activPAL sitting time (p < .01)<br>Group 2: +40 activPAL breaks from sitting/day (p < .01)<br>High intervention satisfaction and adherence	N/A
**Rosenberg** [1]<br>One-arm pre-post pilot over 8 weeks (N = 25; mean age = 71, mean BMI = 34, 28% diabetes, 48% hypertension, baseline activPAL sitting = 9.2 hours/day)	Five phone calls from health coaches, activPAL feedback after baseline and at 4 weeks, goal-setting, problem-solving, motivational interviewing, relapse prevention, cues, self-monitoring	ActivPAL sitting -27 minutes/day, standing +20 mins/day; accelerometer MVPA +24 mins/wk (p's < .05) improved<br>Improved gait speed, depressive symptoms (p's < .05), & weight (p = .07)<br>Among those with SBP > 120, SBP (-6.8 mmHg, p = .06) & DBP (-3.0 mmHg, p < .05) reduced<br>High phone call adherence and study satisfaction	**417643-14**: Take Active Breaks from Sitting Pilot (TABS-P) Study
Rosenberg pilot RCT<br>*Randomized controlled pilot over 3 months (N = 60, mean age =68, mean BMI = 36, 23% diabetes, 60% hypertension, mean baseline activPAL sitting = 10.2 hours/day)*	*Built on findings from our prior study and included 2 in-person sessions, 4 phone calls, activPAL feedback at baseline and 6 weeks, activity band to cue reminders to take breaks from sitting, and home environment audits*	*activPAL sitting (-54 for I-STAND vs. control, p < .05) and standing (+42 minutes/day for I-STAND vs. control, p < .01) improved*<br><br>*Adherence to sessions was 99%, high study satisfaction*	***817486-20:*** *iSTAND R21*
SBP = systolic blood pressure; DBP = diastolic blood pressure; MVPA = moderate-to-vigorous physical activity			

3. Study Design

Describe the overall approach of the study (e.g. prospective, interventional, observational, retrospective, etc.). If your study includes more than one group, arm, or subject population, describe that here (for example, a study of both subjects and their caregivers, or a study with both a prospective interventional arm and a retrospective chart review arm).

We will use a rigorous two-stage RCT design, adapted and refined based on our pilot studies, to test our novel intervention to reduce sitting time in older adults. After baseline measurements, 284 participants will be randomized to the intervention (i-STAND) or an attention control group (Health Lifestyles). After 6 months, i-STAND participants will be re-randomized to receive either booster sessions or no further intervention. Attention control participants will receive no further intervention. All participants will be followed for 12 months total with assessments at baseline, 3 and 6, and 12 months. Our primary outcomes are reduction in sitting time at 6 months, objectively measured using the activPAL device, and blood pressure. Our design allows us to answer novel questions about the impact of sitting reduction on cardiometabolic risk markers as well as maintenance of sitting reduction. I-STAND is designed to be easily implemented within our healthcare system to support practical and scalable approaches to improving cardiovascular health for high-risk older adults.

2022 BUDGET CUT UPDATE: As of March 31, 2022, we are removing Stage 2 from the flow diagram above. Neither the i-STAND cohort nor the control cohort will have a 12-month assessment. Furthermore, the i-STAND cohort will end without re-randomization as previously planned and will not have a subset of participants engaged in booster coaching sessions.

4. Study Population

a. Number of Subjects

State the number (or approximate number, if appropriate) of subjects you plan to include at the KP region to which this study is being submitted. If applicable, distinguish between the number of subjects who are expected to be enrolled/screened and the number of subjects needed to complete the research procedures (e.g. numbers of subjects excluding screen failures).

As appropriate, consider different populations of subjects within the same study (e.g. subject/caregiver, parent/child, patient/physician). If this is a multicenter study, indicate the total number of subjects to be accrued across all sites.

Population	Number to be screened/invited	Number of subjects to be enrolled

KPWA member men and women who are between 60-89	2500-3000*	284*
Original (In-Person) Cohort	-	100
REMOTE Cohort		184
	Total: 2500-3000	Total: 284

b. Inclusion and Exclusion Criteria

- Describe the criteria that define who will be included or excluded in your final study sample.
- Describe how individuals will be screened for eligibility.
- Describe the plan for disposition of data collected during recruitment/screening in the event of a screen failure or when a potential subject is contacted but declines participation (e.g. destroyed immediately, destroyed at end of study, retained for separate analysis or so that subjects are not contacted repeatedly about participation after they have declined, etc.).

Table 2. Inclusion criteria – ORIGINAL (In-Person) **COHORT**

Source	Key Information/Description of Variables
	**Inclusion Criteria:**
KP VDW	• over age 60; BMI ≥30 and <50 kg/m2<br>• men and women of all races and ethnicities<br>• **We will oversample from clinics with greater minority member representation: Capitol Hill, Downtown, Northgate, Rainier, Burien, Renton, Federal Way, Factoria, Bellevue**
KP VDW	no diagnosis codes indicating dementia or serious mental illness (e.g., schizophrenia, bipolar disorder), or a terminal or serious illness (e.g., cancer) or deafness/significant hearing loss in the past 2 years
KP VDW	not residing in long-term care or skilled nursing and not receiving hospice care
	**Phone screening/eligibility variables, such as:**
Participant	• Self-reported sitting time of 6+ hours per day<br>• Able to walk one block, able to speak and read English, no self-reported hearing or vision limitations, sedentary time, use of an assistive device, able to stand from a seated position without help from another person<br>• No cognitive impairment that is perceived by the RS during phone screening

	• Willingness to wear device (activPAL)<br>• Willingness to participate in in-person visits in Seattle<br>• Willing and able to fast for up to 12 hours prior to in-person visit<br>• Ability to participate for a full 12-month period *(i.e.: not planning on moving out of the state and unable to return for 12-month visit)*<br>• No previous participation in either ISTAND or TABS<br>• No current participation in SMARRT

Exclusion Criteria

Exclusion criteria (are intentionally minimal as our intervention is low risk) in order of screening:

1. Evidence of cognitive impairment/dementia/serious mental illness/terminal cancer or other illness including related diagnosis (diagnosis codes – pulled from VDW screening by programmer)
2. BMI less than 30 or greater than 50 (pulled from VDW screening by programmer)
3. Unable to speak and read English (phone screen)
4. Unable to walk 1 block (with or without assistive devices) (phone screen)
5. Self-reported sitting time less than 6 hours per day (phone screen)
6. A record of participation in prior ISTAND or TABS studies (phone screen)
7. Cancer diagnosis within past 12 months
8. Residing in long-term care, hospice care or skilled nursing facility within past 12 months
9. Participants in the ACT study will be excluded from this study. This is necessary to eliminate participant burden due to both studies having lengthy visits and both studies wearing similar devices.
10. Unable to summarize the study purpose and the activities prescribed to the participant after reading through the consent form with the RS at the Baseline visit.

Table 2. Inclusion criteria – REMOTE COHORT

Source	Key Information/Description of Variables
	**Inclusion Criteria:**
KP VDW	• over age 60; BMI ≥30 and <50 kg/m2<br>• men and women of all races and ethnicities from anywhere in the KPWA region (state-wide)<br>• **We will oversample people of color statewide**
KP VDW	no diagnosis codes indicating dementia or serious mental illness (e.g., schizophrenia, bipolar disorder), or a terminal or serious illness (e.g., cancer) or deafness/significant hearing loss in the past 2 years
KP VDW	not residing in long-term care or skilled nursing and not receiving hospice care

	**Phone screening/eligibility variables, such as:**
Participant self-report	• Self-reported sitting time of 6+ hours per day<br>• Able to walk one block, able to speak and read English, no self-reported hearing or vision limitations, sedentary time, use of an assistive device, able to stand from a seated position without help from another person<br>• No cognitive impairment that is perceived by the RS during phone screening<br>• Willingness to wear device (activPAL)<br>• Willingness to receive and use BP monitor and scale<br>• Ability to participate for a full 12-month period *(i.e.: not planning on moving out of the state and unable to return for 12-month visit)* [NOTE FOR REVIEWER: This will remain the 12 month criteria given that baselines will be finished before this change is made]<br>• Not previously enrolled in ISTAND or TABS<br>• Not currently enrolled in ACT, STOP-FALLS or SMARRT studies

Exclusion Criteria
All the same exclusion criteria as the Original Cohort, along with no current participant in the ACT, STOP-FALLS or SMARRT studies as self-reported on the phone screen conducted by they survey research group.

c. Vulnerable Populations
Indicate whether you will include or exclude each of the following special populations. This refers to subjects who are known members of these populations upon enrollment or at any time during the study. Justify the inclusion of any of these populations. Describe additional safeguards to protect the rights and welfare of these subjects.

- Children
- Pregnant Women
- Neonates of uncertain viability or nonviable neonates (up to 28 days post birth)
- Prisoners (NOTE: The KP IRB does not have the appropriate membership to review research involving prisoners. Consultation with KFRI will be required.)

None

d. Identification of Subjects
Describe how you will identify subjects. If you plan to use private records (EHR, Dept. of Licensing) complete 5.e, 5.f., and 5.g below as relevant.

ORIGINAL (In-Person) COHORT:
A KPWA programmer will use KPWA's electronic medical records and other automated data files to identify eligible King County, Washington subjects from virtual warehouse data. (A waiver of consent for this identification method is requested as part of this application.) The programmer will put the variables pulled into a database for recruitment tracking.

Source (specify) (i.e., from KPWA, from an approved or previous study, from another site)	List of electronic data that will be used to **identify** potential subjects.	Date Range (if relevant)
KPWA VDW<br><br>ACT study records	• KPWA members residing in King County<br>• Contact information including phone and address<br>• over age 60 up to age 89;<br>• BMI ≥30 and <50 kg/m^2;<br>• not residing in long-term care or skilled nursing and not receiving hospice care; and,<br>• no diagnosis codes indicating dementia or serious mental illness (e.g., schizophrenia, bipolar disorder), or a terminal or serious illness (e.g., cancer) in the past 2 years or deafness/significant hearing loss.<br>• Systolic blood pressure > 130 two times in previous 12 months (to be used for stratifying at randomization)<br>• Continuously enrolled at KP for previous 12 months<br>• No record of death<br>• Not on the No Contact list<br>• Primary care at Capitol Hill, Downtown, Northgate, Rainier, Burien, Renton, Federal Way, Factoria or Bellevue - Consumer numbers from the ACT study participants will be accessed in order to make sure these participants are not included in the HART study population	Jan 2017-present

REMOTE COHORT:
A KPWA programmer will use KPWA's electronic medical records and other automated data files to identify eligible KP Washington state subjects from virtual warehouse data. (A waiver of consent for this identification method is requested as part of this application.) The programmer will put the variables pulled into a database for recruitment tracking.

Source (specify) (i.e., from KPWA, from an approved or previous study, from another site)	List of electronic data that will be used to **identify** potential subjects.	Date Range (if relevant)
KPWA VDW	• KPWA members statewide<br>• Contact information including phone and address<br>• over age 60 up to age 89;<br>• BMI ≥30 and <50 kg/m^2;<br>• not residing in long-term care or skilled nursing and not receiving hospice care; and,<br>• no diagnosis codes indicating dementia or serious mental illness (e.g., schizophrenia, bipolar disorder), or a terminal or serious illness (e.g., cancer) in the past 2 years or deafness/significant hearing loss.<br>• Systolic blood pressure > 130 two times in previous 12 months (to be used for stratifying at randomization)<br>• Continuously enrolled at KP for previous 12 months<br>• No record of death<br>• Not on the No Contact list	Jan 2017-present

e. Waiver of Informed Consent for **Identification** from EHR or Other Private Records
Provide rationale and justification for the Waiver of Informed Consent for identification of subjects, including:

- Does the proposed research present no more than minimal risk to the study participants?
- How the waiver of informed consent will not adversely affect the rights and welfare of the participants.
- Why this research cannot practically be carried out without a waiver of informed consent.

- Whenever appropriate, the subjects will be provided with additional pertinent information after participation.

A waiver of consent is requested to identify potentially eligible subjects for recruitment. This waiver of consent is required because we do not know who to contact until we access electronic health care data to identify potentially eligible persons. This waiver of consent is required because it would be impractical to contact up to 7000 KPWA patients to obtain their informed consent for us to pull information from their medical chart. This waiver of consent is also requested so we can access consumer numbers from ACT study participants in order to make sure they are not included in our study population.

The primary risk is a breach of confidentiality. Health care benefits and insurance will not be impacted by this screening. No notification will be placed in the patients' EMRs.

With this waiver, we will mail letters to older adult KPWA patients meeting our initial eligibility criteria, offering them an opt-in or opt-out. Those who do not opt-out will be contacted by phone during which time the survey research team member will ask them several screening/eligibility questions, schedule their in-person baseline measurement visit, and notify a research specialist to mail them an activPAL activity monitor to wear for one week prior to the baseline measurement visit.

f. Waiver of HIPAA Privacy Rule Authorization for **Identification** from EHR

If you will not obtain a signed HIPAA Privacy Rule Authorization or if you want to eliminate any required language from the authorization, provide the following rationale and justification.

- Why the research could not practicably be conducted without the waiver.
- Why access to and use of the PHI is necessary for the research.
- Why the use or disclosure of PHI for the research poses no more than minimal risk to the subjects' privacy (must have an adequate plan to protect the PHI from improper use or disclosure, a plan to destroy identifiers at the earliest opportunity consistent with the purpose of the research, and when applicable, written assurances from collaborators that PHI will not be reused or re-disclosed to any other entity).

A waiver of HIPAA authorization is requested to identify potentially eligible subjects for recruitment. This waiver of consent is required because we do not know who to contact until we access electronic health care data to identify potentially eligible persons. This waiver of consent is required because it would be impractical to contact up to 7000 KPWA patients to obtain their consent for us to pull information from their medical chart. This waiver of consent is also requested so we can access consumer numbers from ACT study participants in order to make sure they are not included in our study population.

The primary risk is a breach of confidentiality. The data we screen will never leave KPWA firewall and thus will be well protected.

We will destroy identifiers no later than 6/1/2028.

g. RCW 70.02 Requirements to Access HIPAA Covered Data without Consent for **Identification** from EHR (WA State Law)

If you will not obtain written HIPAA Authorization provide the following rationale and justification. Explain how the research:

- Is of sufficient importance to outweigh the intrusion into the privacy of the patient that would result from the disclosure;
- Is impracticable without the use or disclosure of the health care information in individually identifiable form;
- Contains reasonable safeguards to protect the information from redisclosure;
- Contains reasonable safeguards to protect against identifying, directly or indirectly, any patient in any report of the research project; and
- Contains procedures to remove or destroy at the earliest opportunity, consistent with the purposes of the project, information that would enable the patient to be identified, unless an institutional review board authorizes retention of identifying information for purposes of another research project.

This waiver of consent is required because it would be impractical to screen the data from 7000 KPWA patients to obtain their consent for us to pull information from their medical chart. This waiver of consent is also requested so we can access consumer numbers from ACT study participants in order to make sure they are not included in our study population. Furthermore, this work could not be done with aggregate data only because we need to recruit individuals to participate in an intervention, which requires individual-level data.

All data necessary to complete this research will be created and stored in secure computer files to which only the project staff will have access. Only study staff who are involved in subject recruitment will have access to the files. This includes the research specialists (who will interact with subjects), research survey team members (who will be the first line of contact for the potential subjects), programmer, project manager, and potentially Dr. Rosenberg.

We will destroy identifiers consistent with KPWA consent procedures.

h. Decisionally Impaired Adults

State whether decisionally impaired adults will be included and explain the extent of cognitive impairment (complete, fluctuating, progressive, or temporary). Justify their inclusion, and explain any protections to mitigate risk (such as the involvement of a caregiver or authorized representative). Describe consent/assent procedures.

Not applicable. one of our inclusion criteria is no diagnosis codes indicating dementia or serious mental illness (e.g., schizophrenia, bipolar disorder), or a terminal or serious illness (e.g., cancer) or deafness/significant hearing loss in the past 2 years.

i. Other Populations Targeted for Recruitment

If you are targeting a population that may be vulnerable to coercion or undue influence based on the specific circumstances of the study, describe how you will ensure that participation is voluntary and minimize any added risk. (Common examples include employees, students, people of low socioeconomic status, etc.)
Older adults are often considered a vulnerable population. Older adults are the focus of our study because we believe that by decreasing their sitting time, their overall mental and physical health will be improved. Encouraging people to stand and move more is a low-risk intervention. To protect these older adult participants, the Survey Research Program staff and the study's research specialists will be trained to ensure they clearly describe the study and offer numerous opportunities for questions and clarifications. In addition, we will not recruit participants who have a diagnosis of serious mental health conditions or dementia.

j. <u>Setting</u>
Describe the sites or locations where your research team will conduct the research.

If this is a multi-site study:

- Specify what procedures are being performed at this site or by this site's personnel (consider recruitment, consent process, study procedures, data analysis, etc.).
- State how each site will satisfy its IRB review requirements. Indicate if you are asking this site's IRB to rely on another IRB or if another institution would like to rely on this site's IRB and include this information in the eIRB Initial Project submission

For research conducted outside this site describe: (Community, Reservations etc.)

- Site-specific regulations or customs affecting the research at that location.
- Local scientific and ethical review structure outside this site.

ORIGINAL (In-Person) COHORT:
There will be both phone calls conducted by Survey Research Team members as well as Research Specialists and a Research Interventionist (the health coach), and in-person visits at KPWHRI's research clinic in downtown Seattle, conducted by a Research Specialist. At the baseline visit, the Research Interventionist (the health coach) will also be present. We will have a physician on-call.

<u>By mail and phone:</u>
Potential participants will be mailed a letter describing the study with a phone line to call if they do not wish to participate. Those who do not decline will be called by our Survey Research Program staff. The Survey staff will obtain oral consent to be screened for eligibility by phone, answer any questions about the study, screen for eligibility, and, if eligible, obtain oral consent to wear the activPAL prior to the Baseline visit. Those who consent to participate in this stage will be scheduled by the Survey team for their Baseline measurement session. At 6 and 12 months, participants will receive an activPAL device and questionnaire by mail, and will participate in a follow-up in-person measurement visit (see In Person section below).

For participants in both the intervention i-STAND group (Group A) and the Healthy Lifestyles group (Group B), additional activPAL measurements will be collected (via mailed activPAL) at 3

months; for those *re-randomized* to the i-STAND booster group, they will also receive an activPAL at 9 months via mail. Neither the 3 or 9 month activPAL wears will be followed by an in-person visit. Reminder letters for upcoming in-person visits will be sent one-week prior to visit by the Research Specialist. For each period that an i-STAND subject wears an activPAL device, they will also receive a copy of their feedback chart via mail or in-person (depending on timepoint). If requested by the participant, reminders may be sent by email. Email addresses will be collected on the Contact Sheet if this request is made. Also see Supplement B.

In person:
Written consent to participate in the study will be captured at the in-person Baseline visit. One or two in-person health coaching sessions (depending on cohort). At Baseline, 6, and 12-month in-person visits, measurement data will be obtained. These measurement data include HbA1c via capillary fingerstick blood sample; weight, waist and wrist circumference; blood pressure, physical function, collecting the inclinometer (activPAL), and self-reported survey data. The Research Specialist will provide a beverage and a snack after collecting the finger-stick blood sample. Participants will arrive at the KPWHRI research clinic for the baseline measurement session after an overnight fast of 12 hours (if they are able to), and will meet with the research specialist. Participants will be informed that they are free to withdraw from the study at any time. The consent materials will comply with HIPAA standards and will encourage participants to contact the principal investigator or project manager if they have additional questions.

In addition, we will collect adherence data (tracking completion of the in-person session and phone calls) for both intervention groups as well as additional inclinometer (activPAL) data from the i-STAND intervention group. We will audio-record phone sessions to be used in monitoring treatment fidelity.

COVID-19 Update:
While our in-person visits are restricted due to COVID-19 precautions, we will move to modified 6 and 12-month remote visits so as to continue to collect as much data within the allotted time window for measurement time points. Measurement data collection will be altered to only include weight (self-reported), waist circumference (participants will be mailed a tape measure), blood pressure (participants will be mailed a BP monitor from an outside vendor pending their oral consent), activPAL (participants will continue to be mailed activPALs) and the self-reported survey data. Details included in the Procedures section of this application. We have updated Supplement B so as to enable us to send a link to the REDCap survey via email to participants for whom we have email addresses and who have given us permission to use this as a method of communication.

REMOTE COHORT:
Any participant recruited for remote baseline will be followed remotely for the entirety of the study period.

There will be both phone calls conducted by Survey Research Team members as well as Research Specialists and a Research Interventionist (the health coach). There is a physician on-call available to support the team by phone and email if needed.

By mail and phone:
Potential participants will be mailed a letter describing the study with a phone line to call if they do not wish to participate. Those who do not decline will be called by our Survey Research Program staff. The Survey staff will obtain oral consent to be screened for eligibility by phone, answer any questions about the study, screen for eligibility, and, if eligible, obtain oral consent to wear the activPAL prior to the Baseline visit, as well as oral consent to give their name/address to a third party vendor in order for the study to send devices to the participant (such as a blood pressure monitor). Survey Research staff will also collect email addresses for those who agree to allow us to use email for consent, scheduling and sending program materials. Those who consent to participate in this stage will be scheduled by the Research Specialist team for their Baseline measurement PHONE session. At 3, 6 and 12 months, participants will receive an activPAL device and questionnaire by mail or e-mail, and will participate in a follow-up measurement visit by PHONE.

For Remote-Cohort participants in both the intervention i-STAND group (Group A) and the Healthy Lifestyles group (Group B), the same measurements collected at Baseline , 6 and 12-month visits will also be collected (via mailed activPAL and phone measurement visit) at 3 months. Reminder calls for upcoming phone visits will take place one-week prior to the phone visit by the Research Specialist. For each period that an i-STAND subject wears an activPAL device, they will also receive a copy of their feedback chart via mail or email. If requested by the participant, reminder emails may also be sent by email.

2022 BUDGET CUT UPDATE: As of March 31, 2022, neither the i-STAND cohort nor the control cohort will have a 12-month assessment.

Also by phone:
The RS will obtain written or online consent to participate in the study during a pre-baseline phone call with the participant where they will review and answer questions posed by the participant. If the participant would like to participate after reading through the consent and reviewing over the phone with the RS, the participant will sign the consent form and then return it by mail. The RS will answer any questions about the consent form at this time. If the participant is willing to consent online, after discussing the consent process and reviewing the consent materials, the RS will email a link to a REDCap form with an online consent for the participant to electronically sign. Health coaching sessions by phone will take place starting within 2 weeks of the baseline measurement phone visit. During this baseline phone call, the RS will also collect height and weight to ensure we send them the right size cuff along with their blood pressure monitor to ensure accuracy.

At Baseline, 3 and 6, and 12-month phone visits, measurement data will be obtained. These measurement data include self-reported weight (we will be mailing a scale), waist and wrist circumference; blood pressure (we will be mailing a BP monitor), physical function, collecting the inclinometer (activPAL), and self-reported survey data. We will notify participants via reminder call/email/letter that they should not consume caffeine or tobacco, or exercise, within 30 minutes of their scheduled measurement visit, so as to ensure blood pressure readings are accurate, and we will advise participants that visits will need to be rescheduled if they have had any stimulants prior to the visit. When we schedule their first, baseline visit, we will advise them

off this practice.

We will also ask if they are scheduled to have the COVID-19 vaccine during these scheduled timepoints, as documented side effects from the vaccine such as fatigue or soreness may impact their ability to complete the measurement timepoint. Participants can choose NOT to share this information with us. If they do, we will document the date of the vaccine shot(s) in the database. The consent materials will comply with HIPAA standards and will encourage participants to contact the principal investigator or project manager if they have additional questions.

In addition, we will collect adherence data (tracking completion of the in-person session and phone calls) for both intervention groups as well as additional inclinometer (activPAL) data from the i-STAND intervention group. We will audio-record phone sessions to be used in monitoring treatment fidelity.

2022 BUDGET CUT UPDATE: As of March 31, 2022, neither the i-STAND cohort nor the control cohort will have a 12-month assessment.

k. Recruitment Methods

Describe how study participants will be recruited and enrolled. Indicate whether you will openly recruit using advertisements, websites, or brochures. Indicate if you plan to do targeted recruitment using existing records or referral. (Upload all recruitment materials to your submission to the IRB.)

Describe, by position/title, who will be recruiting and enrolling participants (providing the specific names of research team members is not necessary).

Describe any plans for the participants in the currently proposed study to be re-contacted or recruited for future follow-up studies. (Note that participants should be informed of this potential for re-recruitment during the current study's consent process.)

Using successful, standardized procedures applied in numerous KP studies[22-25] including our own pilot work,[1] a member of the KPWA Survey Research Program will mail recruitment letters describing the study to current KP members who meet preliminary inclusion criteria based on age, BMI, and medical history from KPWA electronic data systems (see inclusion/exclusion section). An opt-out option will be included. Those that are interested can call a study phone line to express interest in learning about the study. In addition, the research specialist will proactively call potential participants who do not opt out of contact one- week after the mailing to describe the study in detail, verify eligibility with a screening questionnaire, obtain verbal consent from those who agree to participate, and schedule the baseline measurement visit (where written consent will be obtained). The Survey Research Program has an excellent track record of recruitment for RCTs.[22,26-28]

ORIGINAL (In-Person) COHORT:

Recruitment

- The study team programmer identifies a recruitment sample based on screening of the electronic medical record (we anticipate a recruitment rate of ~8% based on prior studies) who meet the study criteria.
- The survey research team mails letters describing the study in waves over 3 years. The letter includes an opt-out option if potential participants do not wish to be contacted. Otherwise, within a week of sending the letter, a member of the survey research team will call the potential participant. Participants are also given a study phone line where they can call us to express interest.
- When potential participants are reached by phone for study recruitment, the survey research team member describes the study, answers any questions, and assesses interest and eligibility in participating. If there is interest, the survey research team member obtains oral consent to ask screening/eligibility questions, and asks the questions. If the participant fits our criteria and is interested, the survey research team member will obtain their oral consent to wear the activPAL device (which measures sedentary time) for a week, confirms their participation and schedules an in-person measurement visit. If the participant is not interested, the survey research team will ask if they are willing to provide us with a reason why.
- Research specialists will mail out activPAL device after learning from survey research team that a potential participant has expressed interest in the study and is scheduled for a baseline visit. Approximately 12 days prior to the baseline visit, the device will be mailed. If a participant will not be seen for some time (e.g.: their visit is scheduled two months after phone consent), the research specialist will send an interim letter roughly 2-3 weeks after they phone consent, reminding them of their consent to participate in wearing the device, and let them know to expect a package from the study in the weeks preceding their visit.
- See attached script for phone screening and recruitment, including oral consent.
- Potential participant receives device by mail and follows instructions (see attached) on how to wear the device
- At baseline visit, research specialist will obtain written consent from participant, collect the activPAL device which they wear into their visit, and proceed with gathering baseline visit measurements.

Any ineligibility or refusal to participate information collected will be used for the purpose of investigating lower-than-expected recruitment rates. Note: the only data we will keep and use on those who are ineligible or who refuse to participate are the survey eligibility questions.

REMOTE (Phone) COHORT

Using successful, standardized procedures applied in numerous KP studies[22-25] including our own pilot work,[1] a member of the KPWA Survey Research Program will mail recruitment letters describing the study to current KP members who meet preliminary inclusion criteria based on age, BMI, and medical history from KPWA electronic data systems (see inclusion/exclusion section). An opt-out option will be included. Those that are interested can call a study phone line to express interest in learning about the study. In addition, the research specialist will proactively call potential participants who do not opt out of contact one- week after the mailing to describe the study in detail, verify eligibility with a screening questionnaire, obtain verbal consent from those who agree to participate, and schedule the baseline measurement visit

(where written consent will be obtained). The Survey Research Program has an excellent track record of recruitment for RCTs.[22,26-28]

Recruitment

- The study team programmer identifies a recruitment sample based on screening of the electronic medical record (we anticipate a recruitment rate of ~8% based on prior studies) who meet the study criteria.
- The survey research team mails letters describing the study in waves over 3 years. The letter includes an opt-out option if potential participants do not wish to be contacted. Otherwise, within a week of sending the letter, a member of the survey research team will call the potential participant. Participants are also given a study phone line where they can call us to express interest.
- When potential participants are reached by phone for study recruitment, the survey research team member describes the study, answers any questions, and assesses interest and eligibility in participating. If there is interest, the survey research team member obtains oral consent to ask screening/eligibility questions, and asks the questions. If the participant fits our criteria and is interested, the survey research team member will obtain their oral consent to wear the activPAL device (which measures sedentary time) for a week, their consent to have their name/address given to a third party vendor to receive devices from the study, confirms their participation and is advised to expect a phone call from a Research Specialist within several days to schedule their phone-based measurement visit. If the participant is not interested, the survey research team will ask if they are willing to provide us with a reason why.
- The Survey Research Group will mail out the consent form along with a welcome letter.
- About a week after the consent form has been mailed to the participant, the study team research specialist will confirm that the participant has the consent form in front of them in order to review written consent over the phone, have them sign and mail the form back to KPWHRI. When the form is received, a copy will be made and mailed to the participant to keep for themselves. During this phone call, the RS will also collect height and weight to ensure we send them the right size cuff along with their blood pressure monitor to ensure accuracy.
- Research specialists will mail the activPAL device, and order a blood pressure monitor and scale to be mailed to the potential participant, after learning from survey research team that a potential participant has expressed interest in the study. Approximately 25-30 days prior to the baseline phone visit, the activPAL device will be mailed and the BP monitor and scale ordered. If a participant will not be seen for some time (e.g.: their visit is scheduled two months after phone consent), the research specialist will send an interim letter roughly 2-3 weeks after their phone consent, reminding them of their consent to participate in wearing the device, and let them know to expect a package from the study in the weeks preceding their visit. At this time the RS will also inquire about any scheduled COVID-19 vaccination that may overlap with the forthcoming measurement visit timetable, and remind them to avoid stimulants.
- See attached script for phone screening and recruitment, including oral consent.
- Potential participant receives device by mail and follows instructions (see attached) on how to wear the device and instructions to mail the device back to the Research Specialist

- At the baseline phone visit, measurements will be collected over the phone using the scale and blood pressure monitor sent in advance, with instructions (including avoiding stimulants), and with guidance over the phone. As well as other measurements including height (self-report), waist measurement (self-report using a tape measure that accompanies the scale) and a chair-sit exercise (modified physical function measurement as we are unable to do additional measurements of this nature that would happen in person).

Any ineligibility or refusal to participate information collected will be used for the purpose of investigating lower-than-expected recruitment rates. Note: the only data we will keep and use on those who are ineligible or who refuse to participate are the survey eligibility questions.

RESCREENING
NOTE: As of the clinic/KPWHRI shutdown due to COVID-19, we had recruited 16 people for whom we had scheduled baseline visits in late March and April. These visits were postponed until we could determine how we would proceed with Baseline visits and consenting participants. Because of the changes between the Original Cohort and the Remote Cohort, those 16 individuals who have been waiting to be rescheduled will be contacted using a Rescreening Script (see attached) and screened again using the new, Remote Cohort recruitment screen to ensure they are still (a) interested and (b) eligible to participate now that they will be participating exclusively by phone.

I. <u>Informed Consent Process</u>

Describe how you will obtain and document consent, including:

- Where, when and how the consent process will take place.
- A process to ensure ongoing consent.
- Steps that will be taken to minimize the possibility of coercion or undue influence.
- Any steps that will be taken to ensure the subjects' understanding.
- If you will conduct screening or any other research procedures before obtaining full informed consent, describe this.

ORIGINAL (in-person) COHORT
Potential participants identified via electronic health record screening, will be mailed a letter describing the study with a phone line to call if they do not wish to participate. Those who do not decline will be called by our Survey Research Program staff. The Survey staff will obtain oral consent to be screened for eligibility by phone, answer any questions about the study, screen for eligibility, and, if eligible, obtain oral consent to participate in the study and wear an activPAL device. Those who consent to participate will be scheduled for their baseline measurement session.

At the first visit, the baseline visit, the research specialist will provide the subject with a written informed consent form (see attached) and describe the study to them, answer any questions, and obtain written consent. The RS will also ask the subject to summarize the study to the best of their ability. If they are unable to do so or cannot recall the primary parts of the study (ex: that they have to wear a device periodically; that they will have coaching calls; that they will be randomly assigned to one of two groups), they will be thanked for their time and excused from the study prior to randomization.

As of Modification package 18, the consent form was revised to change the HIPAA authorization to extend the data collection period. An Addendum to Consent form will be used with those participants who have not started using the new consent form which includes added data and med capture information.
COVID-19 UPDATE: For those participants who will have a remote 6 or 12 month visit and need to sign the first Addendum Consent mentioned above that was approved on 9/30/19, we will mail this form to be signed and mailed back.

As of April 2020, we are proposing adding an interview at the 6-month timepoint (see page 23) and as such, are updating the original Consent form for anyone consented once we begin recruitment again, and another Addendum Consent to be mailed to participants (and mailed back).

COVID-19 UPDATE: As of Modification Package 31, we are including an addendum oral consent to permit a continuation of coaching beyond the 6-month period. Due to COVID-19, we are unable to conduct study measurement visits on the schedules planned, and extending coaching contacts will serve to maintain contact with participants who are particularly vulnerable to isolation due to age and promote continued engagement in the study.

The subject will be informed that at any time they can choose to withdraw consent.

Consent will not be documented in their EMR.

Note: in the consent form, we do not describe the standing desk, which is only offered to the i-STAND cohort, because:

1. *This will be offered as a supplemental tool to support the standing intervention, which is the i-STAND intervention and will not be offered to the Healthy Living group*
2. *We do not want those randomized to the Healthy Living group to feel as though they have been given a lesser program simply because they are not given these support* devices (they are being given a different program, that doesn't focus on standing).
3. The desk is not part of the intervention and people are not being required to use it, (neither are they required to use the Fitness device) but mentioning it might create greater disappointment among people assigned to the control arm, leading to differential attrition and undermining our analytic power and scientific integrity of the study. The fact that we observed this in the pilot support this as a realistic scientific concern that needs to be managed appropriately.

COVID-19 UPDATE: As of Package 32, we are also offering an oral consent to participants to permit us to send them a blood pressure monitor via a third party vendor. Oral consent script is included in Package 32.

REMOTE COHORT

Potential participants identified via electronic health record screening, will be mailed a letter describing the study with a phone line to call if they do not wish to participate. Those who do not decline will be called by our Survey Research Program staff. The Survey staff will obtain oral consent to be screened for eligibility by phone, answer any questions about the study, screen for eligibility, and, if eligible, obtain oral consent to participate in the study, wear an activPAL device, and consent to receive devices (such as a blood pressure monitor) by mail from an outside third party vendor. After a positive screen, the survey staff will mail directly to the participant a copy of the consent form along with a welcome letter.

Those who consent to participate will be scheduled by a study Research Specialist for their baseline measurement phone visit. After scheduling the phone visit, the Research Specialist will order the scale (which comes with a tape measure) and the blood pressure device from a third party vendor. the baseline activPAL and instructions on how to use the device, as well as instructions on how to record weight and blood pressure when they receive their scale and blood pressure monitor. We are also including a bio sheet of all team members (previously, only coaches were on this sheet which was distributed by the coach at the baseline coaching session).

During a phone call prior to the baseline phone visit, after the consent form packet has been mailed, the research specialist (after confirming that the participant has the consent form in front of them) will review over the phone with the subject the written informed consent form (see attached) and describe the study to them, answer any questions, and obtain either written consent (where in the form will be mailed back to KPWHRI and a copy will be made and sent back out to the participant ideally within 2 weeks of receipt) or online consent using REDCap, wherein the RS will email a link to the REDCap online consent form for the participant to electronically sign. The RS will also ask the subject to summarize the study to the best of their ability. If they are unable to do so or cannot recall the primary parts of the study (ex: that they have to wear a device periodically; that they will have coaching calls; that they will be randomly assigned to one of two groups), they will be thanked for their time and excused from the study prior to randomization.

The subject will be informed that at any time they can choose to withdraw consent.

Consent will not be documented in their EMR.

Note: in the consent form, we do not describe the standing desk, which is only offered to the i-STAND cohort, because:

1. *This will be offered as a supplemental tool to support the standing intervention, which is the i-STAND intervention and will not be offered to the Healthy Living group*

2. *We do not want those randomized to the Healthy Living group to feel as though they have been given a lesser program simply because they are not given these support* devices (they are being given a different program, that doesn't focus on standing).
3. The desk is not part of the intervention and people are not being required to use it, (neither are they required to use the Fitness device) but mentioning it might create greater disappointment among people assigned to the control arm, leading to differential attrition and undermining our analytic power and scientific integrity of the study. The fact that we observed this in the pilot support this as a realistic scientific concern that needs to be managed appropriately.

m. Waiver of Informed Consent

Provide rationale and justification for the Waiver of Informed Consent for this study, including:

- Does the proposed research present no more than minimal risk to the study participants?
- How the waiver of informed consent will not adversely affect the rights and welfare of the participants.
- Why this research cannot practically be carried out without a waiver of informed consent.
- Whenever appropriate, the subjects will be provided with additional pertinent information after participation.

We are only requesting a waiver of documentation of informed consent for initial screening of potential participants.

n. Waiver of Documentation of Informed Consent

Provide rationale and justification for the Waiver of Documentation of Informed Consent by identifying which of these two conditions applies and how.

1) The research involves no more than minimal risk to participants AND involves no procedures for which written consent is normally required outside of the research context.
2) The signed consent document would be the only record linking the participants to the research, and the principal risk to participants would be potential harm resulting from a breach of confidentiality.

ORIGINAL (in-person) COHORT

We are requesting a waiver of documentation of informed consent for initial screening of potential participants. Oral consent will be obtained by the survey research team prior to asking the screening/eligibility questions and mailing the activPAL. Written consent will be obtained by the research specialist at the subsequent in-person baseline clinic visit.

REMOTE (phone) COHORT

We are requesting a waiver of documentation of informed consent for initial screening of potential participants. Oral consent will be obtained by the survey research team prior to asking the screening/eligibility questions and mailing the activPAL as well as getting consent to give

address/name to a third party vendor for mailing of other devices. Written or online consent will be obtained by the research specialist on a subsequent phone call prior to the baseline phone visit, and if in written form, the participant will mail back their consent form.

o. Alteration of Informed Consent

Identify the required elements of informed consent that you wish to remove or alter. Provide justification for their removal or alteration.

We have created an Addendum to Consent form which will be presented to existing (previously consented) participants at follow-up visits (6 or 12-month depending on where in the timeline they are when this new Addendum is approved by IRB). This is only presented to participants who signed the original consent form which doesn't include the new elements recently approved by the IRB on 9/10/19.

ORIGINAL (in person) COHORT

COVID-19 UPDATE: We have created an Addendum to Consent oral script to offer participants the option to continue coaching past the protocol timepoint as dictated in the original consent form until they are able to complete their remote 6 or 12 month assessments (for those receiving the booster intervention). The majority of participants will only have 10 coaching sessions and a subsection of i-STAND cohort will receive booster sessions. This is to ensure that participants are still working on their study goals up until their outcome measures can be assessed. For those who have already completed a 10th call, the coach will reach out by phone and ask if they are interested in optional additional coaching. If the participant is interested, the coach will use the oral consent process. For those who have not yet had this 10th session and have one forthcoming, the coach will ask at the end of that call if they are interested in optional additional coaching and use the oral consent process.

COVID-19 UPDATE: We have created an Addendum to Consent oral script to offer participants the option of receiving a blood pressure monitor (to keep) which we will send by a third party vendor. The oral consent is to verify their permission to share their mailing address and name with a third party in order to mail the device. Participants can choose not to allow this, in which case we will not collect their blood pressure data in the remote measurement visit.

REMOTE (phone) COHORT

No addendum consents will be implemented at this time with any newly recruited participants into the Remote Cohort. The additional coaching option will not apply to this cohort and the consent to give their name/address to a third party is covered in the screening that occurs during recruitment by the survey research group.

2022 BUDGET CUT UPDATE:

As of March 31, 2022, and upon approval of this IRB submission (Package 58) we will be mailing an Informational Letter to participants in both cohorts advising them of the change to the study and providing them a phone number to call if they have concerns. These letters will be sent in lieu of a revised consent form. In the letter we will highlight:

- Removing the 12-month visit which means $30 less than they originally thought they'd receive for participation
- There is no change to risk
- There will be no potential for additional coaching beyond 6-months (only offered to a subset of the participant population)

p. Non-English-Speaking Subjects

If subjects who do not speak English will be enrolled, describe how the consent discussion will take place and indicate if translated consent forms or short forms will be used. Confirm that an interpreter will assist with the initial consent process and subsequent study visits.

Not applicable

q. Assent of Children and Parent Permission

IMPORTANT NOTE: Consent may be obtained in certain situations. For example, conducting family planning or sexually transmitted disease (STD) research. In addition, for older children ages 16 and up who participate in an adult study, the consent document can be used in place of the assent document.

Describe how you will obtain and document assent/parental permission, including:

- Describe your plan for obtaining parent permission. The permission of one parent is generally sufficient for minimal risk research, or for greater than minimal risk research if there is the potential for direct benefit to the child.
- Note that for studies involving greater than minimal risk with no prospect of direct benefit to the child, permission of both parents is required unless one parent is deceased, unknown, incompetent, or not reasonably available, or when only one parent has legal responsibility for the care and custody of the child.
- Describe whether permission will be obtained from individuals other than parents, and if so, who will be allowed to provide permission.
- Indicate whether assent will be obtained and documented from all, some, or none of the children. If assent will only be obtained from some children (because of very young age, severe cognitive impairment, etc.), indicate which children will be required to assent and which will not.
- When assent of children is obtained, describe whether and how it will be documented.
- When subjects might reach the age of majority during the study, describe the plan to obtain consent from these subjects at that time using an adult consent form.

Not applicable

r. Adults Unable to Consent/Decisionally Impaired

Describe the consent/assent process for Adults Unable to Consent/Decisionally Impaired, including:

- Describe the process to determine whether an individual is capable of consent.
- List the individuals from whom permission will be obtained in order of priority. (E.g., durable power of attorney for health care, court appointed guardian for health care decisions, spouse, and adult child.

- Describe the process for assent of the subjects. Address the following:
    - Whether assent will be required of all, some, or none of the subjects. If assent will be obtained from some subjects, indicate which subjects will be required to assent and which will not.
    - If assent will not be obtained from some or all subjects, an explanation of why not.
    - When assent is obtained, describe how it will be documented.
- Describe the plan to obtain consent if subjects might regain capacity to consent during the study.

Not applicable: one of our inclusion criteria is no diagnosis codes indicating dementia or serious mental illness (e.g., schizophrenia, bipolar disorder), or a terminal or serious illness (e.g., cancer) in the past 2 years.

s. <u>HIPAA Privacy Rule Authorization – if study will use or disclose Protected Health Information (PHI)</u>

Describe the plan to obtain a signed Privacy Rule Authorization from each subject.

HIPAA authorization will get signed with the consent form at the Baseline Visit.

t. <u>Waiver of HIPAA Privacy Rule Authorization</u>

If you will not obtain a signed HIPAA Privacy Rule Authorization, or include HIPAA authorization in the consent form, or if you want to eliminate any required language from the authorization, provide the following rationale and justification.

- Why the research could not practicably be conducted without the waiver.
- Why access to and use of the PHI is necessary for the research.
- Why the use or disclosure of PHI for the research poses no more than minimal risk to the subjects' privacy (must have an adequate plan to protect the PHI from improper use or disclosure, a plan to destroy identifiers at the earliest opportunity consistent with the purpose of the research, and when applicable, written assurances from collaborators that PHI will not be reused or re-disclosed to any other entity).

Not applicable

u. <u>RCW 70.02-Requirments to Access HIPAA Covered Data without Consent</u>

If you will not obtain HIPAA Authorization or Consent provide the following rationale and justification. Explain how the research:

- Is of sufficient importance to outweigh the intrusion into the privacy of the patient that would result from the disclosure;
- Is impracticable without the use or disclosure of the health care information in individually identifiable form;
- Contains reasonable safeguards to protect the information from redisclosure;
- Contains reasonable safeguards to protect against identifying, directly or indirectly, any patient in any report of the research project; and
- Contains procedures to remove or destroy at the earliest opportunity, consistent with the purposes of the project, information that would enable the patient to be identified, unless

an institutional review board authorizes retention of identifying information for purposes of another research project.

Not applicable

5. Study Procedures

Describe and explain the study design, including:

- Procedures to monitor subjects for safety, including who will review the data and at what frequency for safety issues.
- Procedures performed to lessen the probability or magnitude of risks.
- All drugs and devices used in the research and the purpose of their use, and their regulatory approval status.
- The source records that will be used to collect data about subjects. (Attach all surveys, scripts, and data collection forms.)
- What data will be collected including long-term follow-up.
- The duration of an individual subject's participation in the study.
- The duration anticipated to enroll all study subjects.
- The estimated date for the investigators to complete this study (complete primary analyses)

NOTE: It should be clear exactly which procedures will be conducted for the research as opposed to procedures the subjects would undergo (in the exact manner described in the protocol) even if they were not participating in the study.

Describe procedures that will be followed when subjects withdraw from the research, including withdrawal from intervention but continued data collection.

Describe any anticipated circumstances under which subjects could be withdrawn from the research without their consent.

Describe any procedures for orderly termination.

If the study involves genetic testing or collection of genetic information, describe this.

Data, sources, and date range

The following table shows how outcomes data will be collected

Source (specify) (i.e., from KPWA, or from an existing or previous study)	Key Information/Description of Variables	Date Range

activPAL activity monitor	PRIMARY OUTCOME<br>• Change in mean daily sitting at 6 months	Baseline to 6-months
ORIGINAL COHORT:<br>In-person measurement visit at KPWHRI<br>COVID-19 UPDATE AND NEW REMOTE COHORTREMOTE MEASUREMENT	PRIMARY OUTCOME<br>• Change in systolic and diastolic blood pressure	Baseline to 6-months
In-person measurements collected at clinic visit at KPWHRI<br>COVID-19 UPDATE AND NEW REMOTE COHORT: REMOTE MEASUREMENT OF WEIGHT	SECONDARY OUTCOMES<br>• Change in HbA1c (Original Cohort only)<br>• Change in Weight	Baseline to 6 months
In-person measurements collected at clinic visit at KPWHRI<br>COVID-19 UPDATE and REMOTE COHORT: REMOTE MEASUREMENT OF WEIGHT and BP	OTHER EXPLORATORY OUTCOMES for i-STAND BOOSTER ONLY<br>• Change in HbA1c (original cohort only)<br>• Change in Weight<br>• Change in systolic and diastolic blood pressure	Baseline to 12 months
activPAL activity monitor	OTHER EXPLORATORY OUTCOMES for i-STAND BOOSTER ONLY<br>• Change in time spent sitting or lying down<br>• Change in step counts	Baseline to 12 months
Participant questionnaire	OTHER EXPLORATORY OUTCOMES<br>• Change in quality of life<br>• Change in depressive symptoms	Baseline to 6 months

	• Change in physical function	
VDW	Various chronic conditions (e.g. diabetes, cardiovascular conditions); lab values for HbA1c, cholesterol; blood pressure; blood pressure medications; height and weight; medical events (e.g. falls, hospitalizations, heart attack, stroke, blood clots); and, healthcare costs.	One year prior to first Study Visit date to the end of the study for up to 5 years after the study ends, no later than June 1, 2028

2022 BUDGET CUT UPDATE: As of March 31, 2022, neither the i-STAND cohort nor the control cohort will have a 12-month assessment to collect the outcomes listed in the table above. To be as clear as possible - those items have been highlighted in green.

Sequential description
(see attached Study Flow Chart)

ORIGINAL (in-person) COHORT

1. Subjects are contacted by the KPWA survey research team and provide oral consent over the phone in the initial screening and eligibility process; if eligible and interested, subjects will be mailed an activPAL device along with instructions on how to wear it for one week and will be scheduled for their first/baseline visit at KPWHRI research clinic.

2. COVID-19 UPDATE: BASELINE VISITS ARE CURRENTLY ON HOLD Subjects will attend a baseline screening visit at KPWHRI research clinic in downtown Seattle.
   a. At the baseline visit, prior to signing the consent form, the RS will ask the potential participant to recap briefly their understanding of the study. Once subjects have signed written, informed consent at the baseline visit they will have baseline measurements taken, including height, weight, waist circumference, physical function and blood pressure. They will have a small amount of blood collected by finger prick. They will turn in their activPAL device to the research specialist. They will also complete a survey containing self-reported scales regarding health symptoms (depressive symptoms, pain, fatigue), daily activities, and psychosocial mediators (e.g. habit strength, self-efficacy). All participants will receive a $40 incentive for completing measurements at the baseline visit. If participants inquire about their blood values or blood pressure, we will share the information with them but without interpretation and advise them to see their provider if they have questions or concerns.

   b. After baseline activPALs are returned, participants will be randomized (using a link created by the study biostatistician and programmer) to receive i-STAND (N = 142) or healthy lifestyle control (N = 142). To ensure that baseline sitting time is balanced, we will stratify participants into two groups based on self-reported sitting-time using median sitting time from our pilot work as a cut point: moderate (<8.5 hours/day) and high (≥8.5 hours/day). We will also stratify by obesity level (30-35 vs 35+kg/m^2) and

blood pressure (systolic BP over or under 130).

c. After completing measurements, participants randomized to receive the i-STAND intervention condition will meet with a health coach and have an in-person coaching session (at Baseline Visit). During the first intervention session they will review a feedback chart depicting their daily sitting time, standing time, and breaks from sitting from the activPAL they wore for baseline. In the case that the coach encounters difficulty with the STATA software that creates the feedback chart output, coaches will use the raw activPAL data (does not take sleep hours out of the download) and share this with participants. They will explain the data in the same way they would if they were able to produce a feedback chart. Participants in the i-STAND cohort will receive the i-STAND study workbook, self-monitoring forms, and offered a wrist-worn prompting device (Fitness activity band), and a portable tabletop standing podium/desk to be used at home or work or wherever the participant chooses to use it (see i-STAND Workbook for photos and instructions for both items) to cue breaks from sitting. The health coach will provide background information on how sitting could impact their health. The coach will review the feedback chart with the participant, provide reinforcement for times during the week when the participant took more breaks from sitting, assess and enhance participant motivation and uncover values (using motivational interviewing principles[29,30]) to inform developing an action plan to meet graded goals based on participant values. The action plan will include target goals, when/how/where tasks will be performed, strategies to overcome barriers, and how goals map to participant values. Discussion will cover principles of habit formation. The health coach will model taking breaks from sitting by inviting the participant to stand with them at least once during the session.
    - During the in-person session, participants will be instructed in using the wrist-worn activity band and standing desk to help remind them to take breaks from sitting. Our pilot work strongly supported the need for these cues. The band subtly vibrates to prompt a break at preselected intervals (ex: 15, 30 or 60 minutes) and will be set to prompt every 15 minutes to remind them regularly to take breaks from sitting. Because many older adults don't feel comfortable setting up technology and mobile tools, the device will be preset so it functions without syncing to another device. Participants who want to use the activity band smartphone application will receive additional instruction. Study staff will not have access to any data from the device. If any participants feel the frequency (every 15 minutes) is too much, study staff can re-set the band at a lower frequency based on the participants' preference.
    - Those who express interest in other features of the Fitness wrist band will be given a handout showing how to program the device to track other things (ex: steps taken, calories burned), however the study does not track on any of these features/options and the coach will remind the participant that the study is solely interested in whether they find the vibration reminders useful as an outward reminder to stand. *(Usefulness will be measured in a 3-month survey (see section 3a next page).*

d. Participants in the Healthy Lifestyle Control group will meet with a health coach and have an in-person coaching session where they will review the Healthy Living

materials and choose topics of interest to focus on throughout the program. They will learn about how to set clear, actionable goals. They will receive a Healthy Living workbook. The health coach will provide background information and help the participant choose topics of focus to inform developing an action plan to meet graded goals based on participant values. The action plan will include target goals, when/how/where tasks will be performed, strategies to overcome barriers, and how goals map to participant values.

e. All participants will be asked by the health coach during the baseline visit to provide additional contact information. This will be collected on a separate sheet and will be entered into the ACCESS database used by the team.

3. After the initial in-person session:

a. All i-STAND participants will receive one more in-person session and 8 follow-up phone calls (10 contacts total) from their health coach over 6 months. All participants will also be sent an activPAL to wear, as well as a survey, at the mid-point (Month 3) and will receive a $20 incentive for wearing the activPAL at month 3. All participants will receive a reminder phone call from the RS to confirm receipt of the packet containing the activPAL and the survey. If the packet was not received, the RS will resend another packet. At the 3-month mark, health coaches will also collect satisfaction data on use of the devices. This information will be collected over the phone and input into REDCap by the coaches, so as to avoid contaminating the survey data collected by the blinded research specialists, who will be managing the primary survey distribution and data collection.

b. All Healthy Lifestyle Control Participants will receive 9 follow-up phone sessions after their initial in-person session (10 contacts total). Each call will cover a topic of interest to the participant (see Table 4). Relevant content from the Healthy Living workbook will be briefly reviewed and goals will be set. Follow-up calls will check-in with prior calls. The calls will occur every 2-3 weeks over the first 6 months of the study.

c. COVID-19 UPDATE: both i-STAND and Health Lifestyle participants will be offered, using an Addendum to Consent oral agreement, the option of additional coaching calls beyond the 10 sessions to occur on a monthly basis. See page 15 of this protocol for details and see the Addendum Consent for script. 3-month assessments will also still be mailed. A study RS will assemble packets with charged activPALs and participant surveys at their home and mail to participants via the USPS. Study RS has sanitation procedures that are used at the office that will also be used at home in assembling packets (gloved, wipes, etc). Participants will mail the packets back to KPWHRI and a study RS will be checking our mailbox once a week for returned devices/surveys.

d. All participants (i-STAND and Healthy Lifestyles control group) will attend a 6-month measurement visit. A few weeks prior to the visit and to mailing the acctivPAL device to the participants, the RS will call the participant to ensure we have accurate contact information and to remind them of the upcoming timepoint. If the participant has

requested that scheduling visits/reminders of visits be done via email, this information will be sent via email from the RS. The same measurements and assessments will be taken as at baseline except a study satisfaction questionnaire will be included in the self-reported survey. All participants will receive a $40 incentive for completing measurements at the 6 month visit. For those participants who do not show up to their scheduled 6-month visit, we will attempt to reach them by phone and then by mail, encourage their participation and completion of this timepoint. At this in-person visit, if participants inquire about their blood values or blood pressure, we will share the information with them but without interpretation and advise them to see their provider if they have questions or concerns. After completing the 6-month measurement visit, all I-STAND participants will be re-randomized to EITHER receive i-STAND booster sessions (5 additional phone contacts) or no further intervention for 6 months. The health coach will call each participant after they complete their 6 month measurements to inform them of their assignment.

- COVID-19 UPDATE: We will be ordering and sending directly to individual participants from a third party vendor (pending oral consent from participants) Omron BP Monitors (The Omron 3-Series BP7100 model) for any 6 and 12-month measurements. Participants will not be expected to return this device. Instructions will be included in the mailed packet that also includes the survey, activPAL, paper measuring tape (Medline 72” paper measuring tape). RS staff will call participants to conduct the chair-sit measurement and to verbally collect the BP/weight/waist measurements from participants. As usual, study packets mailed to participants will include return envelopes for the activPALs and the surveys.
- We will send incentives to participants when business returns to normal and we are able to send cash incentives. Participants will be notified that they will receive their incentives at a later time.
- We have amended Supplement B to request permission to use email to send the link for the survey (REDCap) to participants for whom we have email addresses and who have given us permission to contact them using email.
- We will track those participants who receive a device from a third party vendor on the internal PHI Disclosure Tracking Form (https://ghriweb01.ghc.org/disclosuretrack/seldisc1.aspx)

e. For those re-randomized into the i-STAND group, 5 booster calls spread over 6 months (about every 6 weeks) will focus on maintaining improvements (or continuing to improve for those who have not met goals), action planning, problem-solving, preventing relapse, and continuing to use previously learned skills and tools.

f. Participants in the i-STAND booster arm will wear the activPAL for an interim week between measurement visits (i.e., at 9 months) and receive an additional feedback chart to help monitor progress towards goals.
COVID-19 UPDATE: Until we return to normal business, this intervention activPAL wear has been temporarily suspended.

g. Participants in the i-STAND booster arm will receive a supplemental survey at 9-month mark to gauge their use of the tools – the standing desk and the Fitness wrist-band. COVID-19 UPDATE: Until we return to normal business, this intervention survey has been temporarily suspended.

4. All participants (i-STAND and healthy living control group) will attend a final, 12-month measurement visit. Prior to the visit they will be mailed an activPAL to wear for a week. The same measurements will be taken as at baseline and at the 6-month visit, including a survey. All participants will receive a $40 incentive for completing measurements at the 12 month visit. i-STAND cohort participants will be allowed to keep their wrist bands and standing desks. Once again, at this in-person visit, if participants inquire about their blood values or blood pressure, we will share the information with them but without interpretation and advise them to see their provider if they have questions or concerns.
COVID-19 Update – see 3c. above for details

5. Shortly after completing the active, 6-month phase of the study, participants will receive a call asking for their oral consent to an interview. After completing the full year in the study including the final, 12-month visit, they will receive a letter thanking them for their participation and inviting them to participate in a final exit interview in the coming months. The letter will provide an opt-out if they do not want to do an exit interview. If we do not hear from the participant, we will assume they are open to receiving an exit interview phone call.).

REMOTE (phone) COHORT

1. Subjects are contacted by the KPWA survey research team and provide oral consent over the phone in the initial screening and eligibility process; if eligible and interested, will be notified to expect a call from a research specialist to schedule their baseline phone visit. Survey research team mails consent form and welcome letter.

2. About 1 week after survey has mailed consent, and up to two weeks before the baseline phone visit, the research specialist (after confirming that the participant has the consent form in front of them) will review over the phone with the subject the written informed consent form (see attached) and describe the study to them, answer any questions, and obtain either written or online consent. If the participant is willing to consent online, after discussing the consent process and reviewing the consent materials, the RS will email a link to a REDCap form (see email script) with an online consent for the participant to electronically sign. If in written form, the participant will mail the form back to KPWHRI and a copy will be made and sent back out to the participant ideally within 2 weeks of receipt. The RS will also ask the subject to summarize the study to the best of their ability. If they are unable to do so or cannot recall the primary parts of the study (ex: that they have to wear a device periodically; that they will have coaching calls; that they will

be randomly assigned to one of two groups), they will be thanked for their time and excused from the study prior to randomization. During this phone call, the RS will also collect height and weight to ensure we send them the right size cuff along with their blood pressure monitor to ensure accuracy.

3. The research specialist will prepare and mail a packet to the potential participant including an activPAL device along with instructions on how to wear it for one week. The research specialist will order a scale (and measuring tape) to be sent to the participant as well as a blood pressure monitor. Instructions will be included. Envelopes for returning the activPAL device are also included.

4. Subjects will participate in a baseline phone visit approximately 30 days after initial screening into the study by the survey research group and roughly 2 weeks after their phone consent.
   a. During the baseline phone visit, participants will either complete a survey containing self-reported scales regarding health symptoms (depressive symptoms, pain, fatigue), daily activities, and psychosocial mediators (e.g. habit strength, self-efficacy) with the research specialist or will complete the survey online (their preference). Participants will be guided through collecting their waist measurement, weight and blood pressure using the devices and tools sent ahead of time. Per instructions and reminders sent to participants, if they have consumed any caffeine or tobacco, or exercised within 30 minutes of the visit, the visit will be rescheduled. Participants will also be guided through the modified chair sit over the phone. All participants will be mailed a $30 cash incentive for completing measurements at the baseline visit. If participants inquire about their blood pressure readings, we will advise them to see their provider if they have questions or concerns.

   b. After baseline activPALs are received by mail, coaches will download the data and participants will be randomized (using a link created by the study biostatistician and programmer) to receive i-STAND (N = 142) or healthy lifestyle control (N = 142). To ensure that baseline sitting time is balanced, we will stratify participants into two groups based on self-reported sitting-time using median sitting time from our pilot work as a cut point: moderate (<8.5 hours/day) and high (≥8.5 hours/day). We will also stratify by obesity level (30-35 vs 35+kg/m^2) and blood pressure (systolic BP over or under 130). Coaches will call participants to notify them of their assigned cohort and to schedule their baseline coaching visit. At this time they will also confirm if they prefer to use email or regular mail.

   c. Participants who were randomized to i_STAND and did not give us their email, will have their first feedback chart mailed to them, along with instructions (see attached) on how to retrieve encrypted emails in the future, in case they prefer to receive their feedback chart by email going forward. All other i-STAND participants who have given us permission to email, will have their charts sent via encrypted email as described in Supplement B. Because it does not require encryption, if the participant has expressed over the phone that they prefer to receive study materials by email, the i-STAND workbook will be sent by email at this time. If not, the notebook will be mailed by USPS. A fitness activity band will also be mailed from a third party vendor and a

standing desk. NOTE: We have amended Supplement B to request permission to send the activPAL feedback chart to i-STAND only participants (as an alternative option to sending them by mail).

d. Within two weeks of completing the phone-based baseline measurements, participants randomized to receive the i-STAND intervention condition will have their first phone coaching session with their health coach. During the first intervention session they will review the feedback chart they were mailed which depicts their daily sitting time, standing time, and breaks from sitting from the activPAL they wore for baseline. In the case that the coach encounters difficulty with the STATA software that creates the feedback chart output, coaches will use the raw activPAL data (does not take sleep hours out of the download) and share this with participants. They will explain the data in the same way they would if they were able to produce a feedback chart. The health coach will provide background information on how sitting could impact their health. The coach will review the feedback chart with the participant, provide reinforcement for times during the week when the participant took more breaks from sitting, assess and enhance participant motivation and uncover values (using motivational interviewing principles[29,30]) to inform developing an action plan to meet graded goals based on participant values. The action plan will include target goals, when/how/where tasks will be performed, strategies to overcome barriers, and how goals map to participant values. Discussion will cover principles of habit formation. The health coach will model taking breaks from sitting by inviting the participant to stand with them at least once during the phone session.

- During the phone session, participants will be instructed in using the wrist-worn activity band and standing desk they were sent, to help remind them to take breaks from sitting. Our pilot work strongly supported the need for these cues. The band subtly vibrates to prompt a break at preselected intervals (ex: 15, 30 or 60 minutes) and will be set to prompt every 15 minutes to remind them regularly to take breaks from sitting. Because many older adults don't feel comfortable setting up technology and mobile tools, the device will be preset so it functions without syncing to another device. Participants who want to use the activity band smartphone application will receive additional instruction. Study staff will not have access to any data from the device. If any participants feel the frequency (every 15 minutes) is too much, study staff can re-set the band at a lower frequency based on the participants' preference.
- Those who express interest in other features of the Fitness wrist band may receive a handout by email or mail showing how to program the device to track other things (ex: steps taken, calories burned), however the study does not track on any of these features/options and the coach will remind the participant that the study is solely interested in whether they find the vibration reminders useful as an outward reminder to stand. *(Usefulness will be measured in a 3-month survey (see section 3a next page).*

e. Participants in the Healthy Lifestyle Control group will have their first phone-based coaching session where they will review the Healthy Living materials, go over in brief the first 5 topics, and advise the participant that after session 5, they can choose topics

of interest to focus on for the rest of the program. They will learn about how to set clear, actionable goals. As with the i-STAND group, if we have their email and their willingness to receive study materials by email, we will email them the Healthy Living workbook. Otherwise this will be sent by mail prior to the first call. The health coach will provide background information and help the participant choose topics of focus to inform developing an action plan to meet graded goals based on participant values. The action plan will include target goals, when/how/where tasks will be performed, strategies to overcome barriers, and how goals map to participant values.

f. All participants will be asked by the health coach during the baseline visit to provide additional contact information. This will be collected on a separate sheet and will be entered into the ACCESS database used by the team.

5. After the initial baseline phone coaching session:

a. All i-STAND participants will receive 9 follow-up phone calls (10 contacts total) from their health coach over 6 months. An optional activPAL wear for i-STAND participants will take place at the 6-week mark, after which their data will be reviewed on a subsequent coaching call.
2022 BUDGET CUT UPDATE: As of March 31, 2022, we are no longer offering the optional 6-week wear as a part of our staff time adjustments

b. All Healthy Lifestyle Control Participants will receive 9 follow-up phone sessions after their initial baseline phone coaching session (10 contacts total). Each call will cover a topic from the workbook (see Table 4). Relevant content from the Healthy Living workbook will be briefly reviewed and goals will be set. Follow-up calls will check-in with prior calls. The calls will occur every 2-3 weeks over the first 6 months of the study.

c. All study participants will be sent an activPAL to wear, as well as a survey, at the mid-point (Month 3) and will participate in a 3-month measurement visit by phone with a research specialist, repeating the measurements and instructions from the baseline visit. If the participant has requested that scheduling visits/reminders of visits be done via email, this information will be sent via email from the RS. They will receive by mail a $30 incentive for completing the 3-month visit. All participants will receive a reminder phone call from the RS to confirm receipt of the packet containing the activPAL and the survey. If the packet was not received, the RS will resend another packet. At the 3-month mark, health coaches will also collect satisfaction data from the i-STAND group on their use of the devices. This information will be collected over the phone and input into REDCap by the coaches, so as to avoid contaminating the survey data collected by the blinded research specialists, who will be managing the primary survey distribution and data collection.

d. All participants (i-STAND and Healthy Lifestyles control group) will participate in a 6-month phone-based measurement visit. If the participant has requested that scheduling visits/reminders of visits be done via email, this information will be sent via email from the RS. The same measurements and assessments will be taken as at baseline and 3-

months except a study satisfaction questionnaire will be included in the self-reported survey. All participants will receive a $30 incentive for completing measurements at the 6 month phone visit. After completing the 6-month measurement visit, all I-STAND participants will be re-randomized to EITHER receive i-STAND booster sessions (5 additional phone contacts) or no further intervention for 6 months. The health coach will call each participant after they complete their 6 month measurements to inform them of their assignment.
2022 BUDGET CUT UPDATE: As of March 31, 2022, there will be no re-randomization at the 6-month timepoint for those in the i-STAND cohort. Their coaching will end after the 10 initial sessions.

e.
- We have amended Supplement B to request permission to use email to send the link for the survey (REDCap) to participants for whom we have email addresses and who have given us permission to contact them using email.
- We will track those participants who receive a device from a third party vendor on the internal PHI Disclosure Tracking Form (https://ghriweb01.ghc.org/disclosuretrack/seldisc1.aspx)

f. For those re-randomized into the i-STAND group, 5 booster calls spread over 6 months (about every 6 weeks) will focus on maintaining improvements (or continuing to improve for those who have not met goals), action planning, problem-solving, preventing relapse, and continuing to use previously learned skills and tools.
g. Participants in the i-STAND booster arm will have the option to wear the activPAL for an interim week between measurement visits (i.e., at 9 months) and receive an additional feedback chart to help monitor progress towards goals.
h. Participants in the i-STAND booster arm will receive a supplemental survey at 9-month mark to gauge their use of the tools – the standing desk and the Fitness wrist-band.
2022 BUDGET CUT UPDATE: As of March 31, 2022, and due to the removal of the re-randomization stage for the i-STAND cohort taking place at 6-months (bullet “f”), there will be no further boosters. At 9-months, there will be no optional 9-month activPAL wear (bullet “g”) nor will there be a 9-month supplemental survey (bullet “h”.

6. All participants (i-STAND and healthy living control group) will participate in a final, 12-month phone-based measurement visit. Prior to the visit they will be mailed an activPAL to wear for a week. The same measurements will be taken as at baseline, 3 and 6 months, including a survey. All participants will receive a $30 incentive for completing measurements at the 12 month visit. i-STAND cohort participants will be allowed to keep their wrist bands and standing desks. Once again, at this phone visit, if participants inquire about their blood pressure, we will advise them to see their provider if they have questions or concerns.
2022 BUDGET CUT UPDATE: As of March 31, 2022, neither the i-STAND cohort nor the control cohort will have a 12-month assessment. As such, there is no $30

incentive for completing a 12-month visit. This will be explained in the informational letter sent to participants.

7. For those participants who miss their scheduled 3, 6 or 12 measurement visits, we will attempt to reach them by phone and then by mail, encourage their participation and completion of that timepoint.

   2022 BUDGET CUT UPDATE: As of March 31, 2022, neither the i-STAND cohort nor the control cohort will have a 12-month assessment

8. Shortly after completing the active, 6-month phase of the study, participants will receive a call asking for their oral consent to an interview. After completing the full year in the study including the final, 12-month visit, they will receive a letter thanking them for their participation and inviting them to participate in a final exit interview in the coming months. The letter will provide an opt-out if they do not want to do an exit interview. If we do not hear from the participant, we will assume they are open to receiving an exit interview phone call.).

2022 BUDGET CUT UPDATE: As of March 31, 2022, we will no longer be conducting exit interviews at the 6-month timepoint. And, as neither the i-STAND cohort nor the control cohort will have a 12-month assessment there will be no exit interview after this timepoint either.

Table 4. Healthy Lifestyle Topics

1. Coping with the Pandemic
2. Insomnia and Sleep
3. Stress & Anxiety
4. Low Mood & Aging
5. Healthy Eating & Hydration
Healthy Bones & Osteoporosis
Arthritis & Healthy Joints
Bladder Control
Preventing Falls
Stress & Anxiety
Writing an Advance Directive
Immunizations & Screenings
Managing your Medicines
Chronic Pain
Emergency Food & Water Supplies
Goal Setting & Tracking Progress
Communicating with Physicians

PARTICIPANT COMPLETION

ORIGINAL COHORT

1. Participants who completed all in-person visits will receive a completion certificate and a

$40 bonus for completing all visits ($180 total).

   a. Participants in both groups may receive the workbook from the condition they did not receive and a summary of their activPAL data at the end of the study if they are interested.

2. Once study results are available, a summary document of study findings will be prepared and shared with all participants.

REMOTE COHORT:

1. Participants who completed all phone visits will receive a completion certificate and a $30 bonus for completing all visits ($150 total).
   2022 BUDGET CUT UPDATE: As of March 31, 2022, there will no longer be a 12-month visit, so the the new total amount is $120. We will continue to give a $30 bonus for completing the other timepoints (Baseline, 3 and 6 months).

   a. Participants in both groups may receive the workbook from the condition they did not receive and a summary of their activPAL data at the end of the study if they are interested.

2. Once study results are available, a summary document of study findings will be prepared and shared with all participants.

ORIGINAL (in-person) COHORT
Additional information about assessments and measurements at in-person visit (baseline, 6 and 12 months):

Cardiometabolic biomarkers: We will measure HbA1c. To measure HbA1c, we will obtain a capillary whole blood sample obtained by finger prick, collected in a toothpick-sized capillary tube, and analyzed immediately using the Siemens DCA Vantage Analyzer. It performs a CLIA-waived HbA1c test that is IFCC and NGSP-certified.

Potential incidental findings may include a discovery that the participant has Diabetes. If this is discovered, the physician on call will notify the research specialist who will advise the participant to meet with their primary care provider as soon as possible.

Weight, Height, Waist. Weight will be measured using a calibrated Seca Digital weight scale. Height will be measured using a calibrated Seca Digital Stadiometer. Waist circumference will be measured twice at the superior border of the iliac crest and the average of two measurements used.[31]
COVID-19 UPDATE: remote weight measurement at 6 and 12-months (until normal business resumes) will be collected as available and depends on the participant having a scale at home. Waist circumference will be collected using a Medline brand paper measuring tape which will be sent ahead of time to participants along with instructions on how to take an accurate measurement.

Blood Pressure: We will measure blood pressure (systolic and diastolic) three times to ensure accurate results. BP will be measured using an OMRON Digital BP Monitor with Intellisense. Model HEM907XL.We will also advise participants to avoid caffeine, tobacco and exercise in the 30 minutes

prior to the visit to ensure accuracy. After consent and when scheduling the baseline visit, we will ask the participant for height and weight so we can plan to send them the right size cuff with their BP monitor.

COVID-19 UPDATE: Until we return to normal business, we will be mailing Omron 3-Series BP7100 monitors (along with cuff and instructions on use) from a third party vendor (pending oral consent from the participant). These were recommended as a good option for home monitoring by Co-I Bev Green, MD who has an expertise in blood pressure. Study measurement of blood pressure using these devices will be conducted during a phone call with the study Measurement RS, allowing for verbal instructions and questions from participants about the proper use of the device.

Demographics: Age, sex, education, marital status, race/ethnicity, retirement status, and other pertinent demographics will be measured using self-reported items on the baseline survey. Some demographic variables will be used as covariates in analyses.

Psychosocial and environmental measures: We will measure self-efficacy based on modifying measures that were developed in youth and adults (since none exist for older adults)[32-35] and habit formation which will be assessed using a modification of the Self-Report Habit Index[36] (e.g., "sitting is something I do without thinking").

COVID-19 UPDATE: We have added new sections to the surveys with validated instruments to better assess participant psychosocial status in the current environment. This includes lower extremity items from the Late-Life Function and Disability Instrument (LLFDI) for physical function; Generalized Anxiety Disorder Assessment (GAD-2) and the Perceived Stress Scale (PSS) for stress/anxiety; and COVID-19 impact. As always, participants can choose not to answer questions on the survey that make them uncomfortable. This information will be used by the statistician to determine whether any of the COVID-19 related variables are moderators of the interventions.

Physical function. The Short Physical Performance Battery (SBBP) will objectively evaluate lower extremity function with tasks for balance, gait speed, and lower-extremity strength (chair rise).[37,38] Gait speed significantly improved in our prior study.[1] SPPB will also be a covariate as it is associated with sedentarism.[39]

COVID-19 UPDATE: Until we resume normal business, we will only be able to measure the chair rise as part of a remote assessment. The study RS will provide instructions and guidance to the participant via phone in order to conduct this measure remotely.

Other behavior changes. We will explore other activPAL metrics including sit-to-stand transitions, standing time, and step counts (as a measure of total physical activity). The same data-processing techniques will be used for these outcomes as for activPAL-measured sitting time. Self-reported sedentary behaviors will be assessed with the Sedentary Behavior Questionnaire to understand what types of sedentary behaviors are amenable to change. This measure has acceptable test-retest reliability and convergent validity.[40]

Quality of life will be measured with the PROMIS Global Quality of Life Scale The Patient Health Questionnaire-8 will assess depressive symptoms and levels improved in our pilot work.[1,41]

REMOTE (phone) COHORT:

Additional information about assessments and measurements at in-person visit (baseline, 3, 6 and 12 months):

2022 BUDGET CUT UPDATE: As of March 31, 2022, neither the i-STAND cohort nor the control cohort will have a 12-month assessment

Cardiometabolic biomarkers: None

Weight, Height, Waist. Weight will be measured using the EtekcityEB9380H scale sent to participants from Amazon. The scale comes with a tape measure. Waist circumference will be measured twice at the superior border of the iliac crest and the average of two measurements used using instructions on how to take an accurate measurement sent by the research specialist.[31]

Blood Pressure: We will measure blood pressure (systolic and diastolic) three times to ensure accurate results. BP will be measured using the Omron 3-Series BP7100 monitors, sent to the participant (along with cuff and instructions on use) from a third party vendor. Study measurement of blood pressure using these devices will be conducted during a phone call with the study Measurement RS, allowing for verbal instructions and questions from participants about the proper use of the device, including to avoid caffeine, tobacco and exercise in the 30 minutes prior to the visit to ensure accuracy.
.

Demographics: Age, sex, education, marital status, race/ethnicity, retirement status, and other pertinent demographics will be measured using self-reported items on the baseline survey. Some demographic variables will be used as covariates in analyses.

Psychosocial and environmental measures: We will measure self-efficacy based on modifying measures that were developed in youth and adults (since none exist for older adults)[32-35] and habit formation which will be assessed using a modification of the Self-Report Habit Index[36] (e.g., "sitting is something I do without thinking"). We have also included validated instruments to better assess participant psychosocial status in the current environment. This includes lower extremity items from the Late-Life Function and Disability Instrument (LLFDI) for physical function; Generalized Anxiety Disorder Assessment (GAD-2) and the Perceived Stress Scale (PSS) for stress/anxiety; and COVID-19 impact. As always, participants can choose not to answer questions on the survey that make them uncomfortable. This information will be used by the statistician to determine whether any of the COVID-19 related variables are moderators of the interventions.

Physical function. We will measure the chair rise sub-scale of the Short Physical Performance Battery as part of the remote assessments. The study RS will provide instructions and guidance to the participant via phone in order to conduct this measure remotely.

Other behavior changes. We will explore other activPAL metrics including sit-to-stand transitions, standing time, and step counts (as a measure of total physical activity). The same data-processing techniques will be used for these outcomes as for activPAL-measured sitting time. Self-reported

sedentary behaviors will be assessed with the Sedentary Behavior Questionnaire to understand what types of sedentary behaviors are amenable to change. This measure has acceptable test-retest reliability and convergent validity.[40]

Quality of life will be measured with the PROMIS Global Quality of Life Scale The Patient Health Questionnaire-8 will assess depressive symptoms and levels improved in our pilot work.[1,41]

Device information
The activPAL (PAL Technologies Ltd, Glasgow, UK) is currently the most valid objective measurement of sitting time as it differentiates sitting from standing using an inclinometer and proprietary software to calculate time spent sitting/lying, standing, stepping, and number of sit-to-stand transitions.[42,43] This device has been used with older adults,[44] is sensitive to change[1,45] and has high validity in comparison to direct observations.[42,46,47] It is considered more valid for measuring sitting time and breaks from sitting than Actigraph or Actiheart accelerometers. [47,48] We will use the activPAL micro which is unobtrusive, very light (10 grams), 5 mm thick, and worn on the thigh using a waterproof medical adhesive (Tegaderm™) that does not require skin preparation such as shaving. The device does not have to be removed. The research specialist will train participants in how to attach and replace the activPAL in the event the dressing becomes compromised or if skin irritation occurs. This training will ensure that I-STAND participants are able to independently apply the activPAL to their leg for interim activPAL wear. The device has worked well in Dr. Rosenberg's sub-study of over 1100 older adults enrolled in Dr. Larson's Adult Changes in Thought Study. Very few reports of difficulties or skin irritation have occurred.

The Fitness activity tracking band will be offered to i-STAND participants (not to the attention control group) for activity prompts at selected intervals. Participants can choose at any time to not use the band (some may not find the reminders useful). No data will be collected from the activity band itself. It is solely used as a prompting device for 15-minute reminders to stand.

Direct identifiers that will be collected or included in the analytic data files, the linking file, or other study records (e.g., recruitment records, intervention files) at KP Washington.

DIRECT IDENTIFERS		
☒ Names	☐ Social Security Numbers	☐ Web URLs
☒ Dates	☒ Medical record numbers	☒ IP address numbers
☒ Postal address	☐ Health plan numbers	☐ Biometric identifiers (e.g., finger prints, voice prints, retina scans)
☐ Geocode	☐ Account numbers	☐ Facial Photos/Images
☒ Phone numbers	☐ License/Certificate numbers	☒ CHS ID
☐ Fax numbers	☐ Vehicle ID numbers	☐ Other unique identifier(s):
☒ Email address	☐ Device identifiers/Serial numbers	

Attention Control group:
ORIGINAL (in-person) COHORT:

This intervention was designed to control for a similar amount of contact time and attention, nonspecific treatment effects, study satisfaction, and patient expectations. Attention controls have been used in previous physical activity intervention trials among older adults for whom time, attention, and social support may be particularly salient factors that affect study outcomes.[49,50] Attention control participants will receive the same number of contacts as i-STAND participants and a study workbook with general healthy living content based on the usual care available to KP members. During the initial in-person session, participants will choose up to 9 topics from a list of 17 to review with their health coach during subsequent sessions (see Table 2). The content provided will not include any information that could impact sitting time or physical activity. During the 9 follow-up calls, the health coach will review progress towards prior goals, engage participants in brief discussions of the session topic, and set goals for new topics. All health-related content included in the workbook and delivered by the health coach is currently available online to KP members and is reviewed and updated regularly by KP physicians. The attention control group will receive no further intervention after the 6-month measurement visit.

REMOTE (phone) COHORT:
This intervention was designed to control for a similar amount of contact time and attention, nonspecific treatment effects, study satisfaction, and patient expectations. Attention controls have been used in previous physical activity intervention trials among older adults for whom time, attention, and social support may be particularly salient factors that affect study outcomes.[49,50] Attention control participants will receive the same number of contacts as i-STAND participants and a study workbook with general healthy living content based on the usual care available to KP members. During the initial phone session, participants will review with their coach the first 5 topics (topics for Sessions 1-5 are assigned to the participant). After the first 5 sessions, the participant can choose and additional 5 topics that interest them for Sessions 6 – 10 (see Table 2). The content provided will not include any information that could impact sitting time or physical activity. During the 9 follow-up calls, the health coach will review progress towards prior goals, engage participants in brief discussions of the session topic, and set goals for new topics. All health-related content included in the workbook and delivered by the health coach is currently available online to KP members and is reviewed and updated regularly by KP physicians. The attention control group will receive no further intervention after the 6-month measurement visit.

Blinding (incomplete disclosure): Health coaching training, fidelity, and tracking.
Health coaches will be trained and supervised by Dr. Rosenberg, a licensed clinical psychologist who has training in motivational interviewing, cognitive behavioral therapy, acceptance and commitment therapy, and behavioral activation. Training *for coaches* will include 8 hours of didactic information on principles of coaching, motivational interviewing, and the behavioral strategies to be employed in each condition. Coaches will receive detailed intervention phone scripts *for each condition* to enhance fidelity to intervention content. Coaches will undergo thorough practice of in-person and phone sessions including a final evaluation with attention control or i-STAND practice patients. To enhance fidelity, all phone calls will be audio-recorded and a random 10% will be reviewed by Dr. Rosenberg for content

coverage using a study-specific checklist. *Any deviations from protocol will be rectified during health coach supervision which will occur weekly (separately).* Participants identified as having any concerning physical or mental health issues will be called by Dr. Rosenberg or Drs. Arterburn or Green, the study physicians, who will seek participant permission to notify the participant's primary care physician of the concerns through the KPWA secure messaging system. All participant intervention contacts will be tracked using a tailored Microsoft Access database developed by a programmer. The Health Coaches will not be blinded. Only the research specialist will be blinded. The blinded research specialist who conducts measurements will not have access to the intervention tracking database.

As described on page 12 of this protocol, there is some blinding/incomplete disclosure to participants during the consent process.

Procedures to monitor, lessen or mitigate risk
Use of KP Consulting Nurse Service as needed
Physician on call
Participants will be fully informed about the study
Risk of breach of privacy described further in section 5.f.
Risk of breach of security described further in section 7.
Data Safety Monitoring Plan in place: See DSMP for protocols around concerning health (mental and physical) risks as well as elevated BP, HbA1c.

Subject drop out or withdrawal procedures
ORIGINAL (in-person) COHORT:
At any time, subjects can opt out of the study. Anyone who wishes to drop out will be called by their health coach to determine if they are still willing to provide measurement data, and a letter will be sent relevant to the timepoint in question. A participant may be dropped from the study if they state during the course of the study that they can no longer participate in calls and/or visits (for instance if they move). Participants may also be dropped if at some point their health has deteriorated to a point where their participation is no longer optimal (we are recruiting participating up to age 89 so some decline in general health as a result of age may occur during the year-long participation. This will result in the participant no longer being able to participate. This determination will be made by the PI. This is also described in the consent form.

REMOTE (phone) COHORT:
At any time, subjects can opt out of the study. Anyone who wishes to drop out will be called by their health coach to determine if they are still willing to provide measurement data, and a letter will be sent relevant to the timepoint in question. A participant may be dropped from the study if they state during the course of the study that they can no longer participate in calls. Participants may also be dropped if at some point their health has deteriorated to a point where their participation is no longer optimal (we are recruiting participating up to age 89 so some decline in general health as a result of age may occur during the year-long participation. This will result in the participant no longer being able to participate. This determination will be made by the PI. This is also described in the consent form.

Anticipated study end date
We anticipate completing all subject visits/phone calls by June 30, 2022. COVID-19 UPDATE: We anticipate this date extending to December 2022. We have notified our NHLBI program officer.

a. Data Analysis

Describe the data analysis plan, including:

- Statistical procedures.
- When applicable, the power analysis.
- Any procedures that will be used for quality control of collected data.

FOR BOTH ORIGINAL AND REMOTE COHORTS: We will add secondary analyses to address changes due to COVID-19.

Preliminary analyses. The statistician will be blinded to intervention condition and intent-to-treat analyses will be performed. Before analysis, data will be cleaned and checked for out-of-range and invalid values. We will examine the distribution of baseline covariates across groups to evaluate balance by randomization assignment. Further, we will compare follow-up rates by group assignment along with assessing for imbalance of baseline covariates by intervention group in the subset with follow-up outcome measures. Given the large sample size, we expect that between-group differences will be small. However, if factors differ by group at baseline, we will adjust for them in the final analyses or use them in imputation analyses if we have a larger than expected dropout rate. All analyses will be based on the “intent-to-treat” principle. Regardless of actual intervention participation, outcomes will be analyzed using the group to which participants were randomly assigned. We will also attempt to obtain follow-up data from all participants, regardless of participation level.

Aim 1: Determine the efficacy of i-STAND to improve primary and secondary outcomes at 6 months.

Primary Outcome Analysis: Our primary outcomes are change from baseline in mean daily sitting time *and systolic and diastolic blood pressure* (described in Section C12). We will compare group means for our primary outcomes between participants randomized to i-STAND versus attention control using linear regression with generalized estimating equations (GEEs) including both outcome time points at 3 and 6 months. GEE is used to account for multiple outcomes measured on the same study participant along with relaxing the normality assumption that standard linear regression methods require.[51] We will adjust for baseline covariates including participant age, sex, physical function, and respective baseline outcomes with other covariates that are imbalanced in preliminary analyses. If dropout is above 15%, we will employ imputation techniques to address missing data issues.[52] However, our focus will be on minimizing dropout rates. Analyses for secondary outcomes including lipids, fasting glucose, weight, and waist circumference will be similar, adjusting for respective baseline outcomes in the analysis. Aim 2: Determine the maintenance of sitting and blood pressure reductions over 12 months. We will conduct a sequential series of analyses using a similar statistical framework as outlined for Aim 1, but using 12-month follow-up data. We will first run a regression model with three groups (attention control, i-STAND no boosters, and i-STAND with boosters) including baseline covariates as outlined above. We will then

assess differences in primary outcomes at 12 months in the two i-STAND groups: with and without the booster intervention. If a significant difference in a given primary outcome is found in sitting time at 12 months between the i-STAND booster versus no booster groups (Scenario 1), we will further compare the i-STAND booster and no booster groups to the attention control group. Scenario 1 assessments will determine if i-STAND with boosters is better than i-STAND without boosters and if either or both I-STAND groups are better than attention control at 12 months.

If i-STAND groups do not differ at 12 months (Scenario 2), we will combine i-STAND groups and run a second regression model on each 12-month outcome including only attention control and i-STAND. If this regression shows that i-STAND has lower sitting time and/or blood pressure than attention control, we will conclude that the i-STAND intervention improved sitting time and/or blood pressure over 12 months, but boosters were not shown to be efficacious. We will conduct a similar analysis for secondary cardiometabolic risk marker outcomes.

2022 BUDGET CUT UPDATE: As of March 31, 2022, neither the i-STAND cohort nor the control cohort will have a 12-month assessment, so we are removing Aim 2. However, we will still explore maintenance of sitting and blood pressure reductions over 12-months for future grants and pilot data purposes.

Aim 3: Explore moderators (e.g., age, sex, baseline physical activity) and mediators of reductions in sitting time (e.g., psychosocial variables) and cardiometabolic risk factors (e.g., changes in sitting time and/or physical activity). Exploratory analyses will assess moderators of the intervention effect by including interactions with baseline variables such as age, sex, and physical activity level to determine if a subset of the population might be the best target for dissemination of the i-STAND intervention. If i-STAND is efficacious in reducing sitting time and/or blood pressure, we will also conduct mediator analyses[53] to explore whether psychosocial or behavioral variables mediate the reduction in sitting time and/or blood pressure. All mediator analyses will only include mediators that are measured at time points prior to the outcome time measurement (e.g., 3-month mediators for 6-month outcomes) to assess for causal mediation relationships. We will take a forward stepped approach to assess 1) if a potential mediator is a mediator without including other potential mediators (bivariate mediator), and then 2) a multiple mediator analysis including all bivariate mediators in the model.[54] This will allow us to assess if the mediation is independent of, or in combination with, other mediators. Finally, if i-STAND is efficacious in changing secondary cardiometabolic risk factors, we will explore whether reduced sitting time mediated these changes independent of increased physical activity. This analysis will provide important novel information on whether reduced sitting, regardless of changes in physical activity, can truly improve health among obese older adults.

Additional exploratory analyses: We will explore whether i-STAND improves standing time, breaks from sitting, physical activity (step counts), quality of life, physical function, and depressive symptoms. Further, per protocol analyses amongst those that adhered to the intervention will also be conducted. These analyses will use similar models as described for Aim 1 analyses.

Power calculations. Our sample size of 284 ensures 90% power to detect a difference in the reduction of sitting time between i-STAND and attention control of 41 minutes at 6 months (primary time point). Given our pilot results and i-STAND's long-term goal target of a 2 hour reduction in sitting time, a difference in sitting time reduction of 41 minutes is achievable. We assumed a standard deviation (SD)

in change in sitting time of *97 minutes/day based on I-STAND pilot RCT data*, a pooled sample t-test, and a 15% loss to follow-up under which we would observe 241 participants at 12 months. *Further, this sample size provides 90% power to detect a difference of 6.2 mmHg change in systolic (14.7 SD) and 4.0 mmHg change in diastolic (9.5 SD) blood pressure. We are powered for comparable reductions in blood pressure as those observed in walking and exercise[55,56] interventions (5-10 change in systolic and 1-6 mmHg change in diastolic blood pressure)[57]. Therefore, we are powered to achieve a minimal clinically important difference for blood pressure outcomes.[58]*

For Aim 2, we will have 90% power to detect a 58-minute difference in sitting time between i-STAND with boosters compared to i-STAND without boosters at 12 months. Further, given Scenario 2 in which i-STAND boosters is not different than i-STAND without boosters we will have 90% power to detect a 41-minute difference in sitting time with combined I-STAND groups. Given Scenario 1, in which i-STAND with boosters is significantly different from i-STAND without, at 12 months, we will have 90% power to detect a 50-minute difference in sitting time between either i-STAND group and the attention control. *For blood pressure outcomes, we have 90% power to detect an 8.8 mmHg change in systolic blood pressure and 5.7 mmHg change in diastolic blood pressure when comparing i-STAND groups (booster vs. no booster) at 12 months*. Therefore, this study is well powered to address all aims.

2022 BUDGET CUT UPDATE: As of March 31, 2022, for Aim 2, assuming we have 124 participants completing 12-month outcome follow-up, we will have 90% power to detect a 81-minute difference in sitting time between I-STAND with boosters compared to I-STAND without boosters at 12 months. Further, given Scenario 2 in which I-STAND boosters is not different than I-STAND without boosters we will have 90% power to detect a 57-minute difference in sitting time with combined I-STAND groups. Given Scenario 1, in which I-STAND with boosters is significantly different from I-STAND without, at 12 months, we will have 90% power to detect a 70-minute difference in sitting time between either I-STAND group and the attention control. For blood pressure outcomes, we have 90% power to detect an 12.3 mmHg change in systolic blood pressure and 8.0 mmHg change in diastolic blood pressure when comparing I-STAND groups (booster vs. no booster) at 12 months. We acknowledge we will likely be underpowered for Aim 2 and this is why we have now made it exploratory.

b. Sharing of Results with Subjects

Describe whether results (study results or individual subject results, such as results of standard or research lab tests and genetic tests) will be shared with subjects or their providers.

If the study carries a risk of incidental findings, describe your plan for evaluating these and determining whether and how subjects or their providers will be given this information.

If laboratory results will be shared with subjects or their healthcare providers, verify that the laboratory conducting the test is Clinical Laboratory Improvement Amendments (CLIA) certified.

ORIGINAL (in-person) COHORT:
Summary of overall study findings at completion of study will be shared with participants. During the course of the study, only the i-STAND group will receive feedback charts (showing their

activPAL data). Both groups may receive materials from the group they were not randomized to if they request. Neither group will receive printouts of their HbA1c (or blood glucose*) levels as these are for data purposes only and are not clinically relevant. As noted in the description of the visit procedures, if participants inquire about their blood values or blood pressure, we will share the information with them but without interpretation and advise them to see their provider if they have questions or concerns. If there are abnormal results, as stated in the consent form, "…if results are out of range, we will contact the study physician on call and confirm that there are no irregularities of concern. You may be advised to contact your primary care provider after the visit if there is a concern."

(*This applies to those who previously had blood glucose tested prior to removal of this as a measurement item in early 2020.)

REMOTE (phone) COHORT:
Summary of overall study findings at completion of study will be shared with participants. During the course of the study, only the i-STAND group will receive feedback charts (showing their activPAL data). Both groups may receive materials from the group they were not randomized to if they request. As noted in the description of the visit procedures, if participants inquire about their blood pressure, we will advise them to see their provider if they have questions or concerns. If there are abnormal results, as stated in the consent form, "…if results are out of range, we will contact the study physician on call and confirm that there are no irregularities of concern. You may be advised to contact your primary care provider after the visit if there is a concern."

c. Data and/or Specimen Banking

Indicate if specimens may be used for future research and whether that may include genetic research.

State if data or specimens will be sent to a separate repository. If data or specimens will be banked in a repository for future use as part of this protocol submission address the following questions:

- What will be banked and what identifiers will be associated with the data or specimens?
- Where and how will the data or specimens be stored?
- For what purpose will the data or specimens be used?
- How will the data or specimens be accessed, and who will have access?
- Describe the procedures to release data or specimens, including the process to request a release, approvals required for release, who can obtain data or specimens, and the data to be provided with specimens.

Not applicable

6. Privacy, Confidentiality and Data Security

Describe the steps that will be taken to protect subjects' privacy during recruitment, consent and study procedures.

ORIGINAL (in-person) COHORT:

- Letters: Letters will be addressed only to participant with "confidential" marked clearly on envelope

- Emails will be sent if requested by the participant for the purposes of scheduling a visit/coaching call or sending a reminder about a visit/coaching call. Any emails sent with program materials will include a reminder not to discuss personal information in a response. If a feedback chart is emailed by request to an i-STAND participant, it will be sent via encrypted email and participants will be given instructions (included in this IRB mod) on how to decrypt that email.
- Phone calls will only occur with those participants who have not opted out of being reached (per their letter, they will be provided with an opt-out number). If the participant is not home, staff member will call again when they can speak with participant.
- In-person written consent will take place in the privacy of a clinic room at the downtown Seattle Kaiser Research Institute
- All in-person sessions will be private (not group-based)
- Study participation will not be documented in EMR*
- File destruction will occur no later than 5 years from the end of the study or June 1, 2028.

* If a participant is found to have out-of-range blood pressure or blood draw readings during a measurement visit, the RS may contact the staff MD on call and/or the KP Consulting Nurse Service. In this event, the nurse service may make a note in the participant's medical record of the out-of-range reading. If the MD on call determines the measurement is of particular concern, s/he may notify the participant's provider as outlined on page 3 of the study DSMP.

REMOTE (phone) COHORT:

- Letters: Letters will be addressed only to participant with "confidential" marked clearly on envelope
- Emails will be sent if requested by the participant for the purposes of scheduling a visit/coaching call or sending a reminder about a visit/coaching call. Any emails sent with program materials will include a reminder not to discuss personal information in a response. If a feedback chart is emailed by request to an i-STAND participant, it will be sent via encrypted email and participants will be given instructions (included in this IRB mod) on how to decrypt that email.
- Phone calls will only occur with those participants who have not opted out of being reached (per their letter, they will be provided with an opt-out number). If the participant is not home, staff member will call again when they can speak with participant.
- All phone sessions will be private (not group-based)
- Study participation will not be documented in EMR*
- File destruction will occur no later than 5 years from the end of the study or June 1, 2028.
- If using e-consent: The completed PDFs are in the REDCap File Repository (located behind KP firewall) under "PDF Survey Archive." Files can be downloaded as individual records or bundles in a ZIP file. Note: only users with 'Full data set' data export privileges will be able to download the archived files. The e-Consent Framework also records the IP address of the participant and displays this information in the file repository in order to help regulate potential duplicate forms from a single IP address. PDFs will be downloaded to the G drive so that we can retain copies per the study for up to 5 years. When the study ends, we will deleted the REDCap files.

* If a participant is found to have out-of-range blood pressure during a measurement visit, the RS may contact the staff MD on call and/or the KP Consulting Nurse Service. In this event, the nurse service may make a note in the participant's medical record of the out-of-range reading. If the MD on call determines the measurement is of particular concern, s/he may notify the participant's provider as outlined on page 3 of the study DSMP.

a. Describe the plan for storage of data and/or specimens.
   - Who will have access and how.
   - Where the data/materials will be stored and for how long.
   - What identifiers will be included.
   - Any other steps that will be taken to ensure security (e.g., training of staff, authorization of access, password protection, encryption, physical security, and separation of identifiers from data and specimens, certificates of confidentiality).
   - Describe the plan to deidentify data, destroy data, or retain/archive data at the end of the study.

Data Security: Only study staff that need to know will be able to access identifiable information about the human subjects. This includes research support specialists working with subjects, project managers, Dr. Arterburn, Dr. Green and Dr. Rosenberg, and health coaches. All data containing identifying information will be kept on password-protected computers using password-protected files. Outcomes data will be stored in a separate file for data analysis and no personally identifiable information will be kept in that file; instead we will use unique study identifiers.

In addition, we will collect adherence data (tracking completion of the in-person session and phone calls) for both intervention groups as well as additional inclinometer (activPAL) data from the i-STAND intervention group. We will audio-record phone sessions to be used in monitoring treatment fidelity.

The likelihood of breach of confidentiality is minimal, as outcome-oriented study data are identified only by study ID and files are password protected. Hard copies, audio files, and tracking data are retained in secured locations accessible only to project staff with a specific need for access. All KPWHRI staff receive training in the protection of confidentiality of research participants. Participants will be informed of all anticipated risks in the informed consent and provided with verbal and written safety information.

Multiple steps will be taken to protect participant confidentiality. Subjects' personal identities will not appear on any study data forms, and all forms and data files containing personal identifiers will be stored separately in secured files. All study files, including digital audio files of the intervention sessions, will be maintained in a centralized location on the KPWHRI departmental server. Access to the file is password protected and subject to the same security protections as other confidential health plan data. Access to the audio files will be limited to the study health coaches and Dr. Rosenberg who will review the tapes for treatment fidelity. Audio files will be deleted as soon as possible after they are coded for fidelity. All staff are trained in appropriate

security protections, computer passwords are changed on a regular basis, and all staff sign annual confidentiality agreements.

i-STAND participants who use Fitness devices will not have those devices linked to a personal account. They will be solely linked to a study-account for the purpose of setting the 15-minute reminders to stand. No PHI will be linked to the device.

b. Collection of data from subjects electronically

If you will collect any data from participants electronically (including email, website, etc.), explain:

- How the data will be collected.
- How the information will be secured (encryption, password protection, etc.; may require consultation with IT department).
- Any risks to the participants' privacy posed by using these methods (describe in consent, as applicable).
- How you will verify the participant's identity.

ORIGINAL (in person) COHORT:

Data Collection:
Electronic data collected as part of this study will include:

1) Email addresses will be collected if a participant indicates that they wish to manage scheduling, consent or reminders for visits and calls via email.

2) Self-reported survey results, documented in REDcap (input either directly by participants using a tablet or, if participant is not comfortable with the technology, they can provide the responses to the survey questions on paper and it will be entered by the research specialist; the paper copy will be destroyed. Note: we are using an internal version of the REDcap database that exists behind the KPWA firewall which also applies to those consents to be collected online. The completed signed (consent) PDFs are temporarily stored in the REDCap File Repository under "PDF Survey Archive." Only study staff with full access to the REDCap account have access to these files. They will be downloaded to a G Drive folder so we can delete the entire REDCap project once the study is closed. As with other study files, these will be destroyed five years after the study ends as noted throughout this document

3) Data provided by the inclinometer (activPAL) worn by participants at key timepoints.

Data from the activPAL is collected by taking the small device, inserting a connecting cord from the device to a KPWHRI computer with activPAL software, downloading the participant's activity record from the previous week and using that record to provide an visual of how much

sedentary time occurred. This will all be done by a research specialist who will receive training in using the activPAL software. The activPAL device and the software for analyzing results are both made by Glasgow, Scotland-based PAL Technologies Ltd.

i-STAND group participants will be asked to wear activPAL activity monitors for five or six (if they get boosters) 1-week periods: baseline, one week after baseline, one week at 3 months post-randomization, one week at midpoint (6 months), one week at 9 months (those receiving boosters only) and at 12 months (follow-up). Healthy living group participants will be asked to wear activPAL activity monitors for four 1-week periods: baseline, 3 months, 6 months, and 12 months (follow-up).

We will also give i-STAND participants the option of wearing activity wristbands and using a portable standing desk. The wristbands provide a mild vibration after 15 minutes of inactivity. Settings will be configured using a generic study team account with no identifiable patient data; participants will not create their own account and no personally identifiable information will be shared with the company making the activity bands, as all bands will be assigned a generic email unattached to a subject name or identification.

Participants can stop wearing the activPAL and activity devices at any time.

Once an activPAL device is returned by a participant, all data from the activPAL devices will be promptly downloaded directly onto KPW computers by a study RS. It will never be stored or shared outside the KP firewall. Data from the activity device will not be downloaded or stored for any reason. Upon return of the device, a study RS will wipe the device, resetting it for future use.

COVID-19 UPDATE: Until normal business resumes, activPAL data will be downloaded onto study team member home computers (coaches and RS staff). All staff member personal computers are password protected. In addition, the activPAL data is fully de-identified. Once downloaded from the activPAL device, data will be immediately moved to the KPWHRI shared drive and deleted from personal computers.We have completed a Tech Risk Review which deemed this activity from home, using Secure File Transfer from a home computer to a KPWHRI shared drive is considered low risk.

REMOTE (phone) COHORT:
Data Collection:
Electronic data collected as part of this study will include:

4) Email addresses will be collected if a participant indicates that they wish to manage scheduling and reminders for visits and calls via email.

5) Self-reported survey results, documented in REDcap (input either directly by participants using a tablet or, if participant is not comfortable with the technology, they can provide the responses to the survey questions on paper and it will be entered by the research specialist; the paper copy will be destroyed. Note: we are using an internal version of the REDcap

database that exists behind the KPWA firewall.

6) Data provided by the inclinometer (activPAL) worn by participants at key timepoints.

Data from the activPAL is collected by taking the small device, inserting a connecting cord from the device to a KPWHRI computer with activPAL software, downloading the participant's activity record from the previous week and using that record to provide an visual of how much sedentary time occurred. This will all be done by a research specialist who will receive training in using the activPAL software. The activPAL device and the software for analyzing results are both made by Glasgow, Scotland-based PAL Technologies Ltd.

i-STAND group participants will be asked to wear activPAL activity monitors for five or six (if they get boosters) 1-week periods: baseline, an optional one week wear 6 weeks after baseline, one week at 3 months post-randomization, one week at midpoint (6 months), another optional one week wear at 9 months (those receiving boosters only) and at 12 months (follow-up). Healthy living group participants will be asked to wear activPAL activity monitors for four 1-week periods: baseline, 3 months, 6 months, and 12 months (follow-up).

2022 BUDGET CUT UPDATE: As of March 31, 2022, neither the i-STAND cohort nor the control cohort will have a 12-month assessment. Furthermore, the i-STAND cohort will not be offered the optional wears (6-week and again at 9-months if they were to be re-randomized).

We will also give i-STAND participants the option of wearing activity wristbands and using a portable standing desk. The wristbands provide a mild vibration after 15 minutes of inactivity. Settings will be configured using a generic study team account with no identifiable patient data; participants will not create their own account and no personally identifiable information will be shared with the company making the activity bands, as all bands will be assigned a generic email unattached to a subject name or identification.

Participants can stop wearing the activPAL and activity devices at any time.

Once an activPAL device is returned by a participant, all data from the activPAL devices will be promptly downloaded directly onto KPW computers by a study RS. It will never be stored or shared outside the KP firewall. Data from the activity device will not be downloaded or stored for any reason. Upon return of the device, a study RS will wipe the device, resetting it for future use.

COVID-19 UPDATE: Until normal business resumes, activPAL data will be downloaded onto study team member home computers (coaches and RS staff). All staff member personal computers are password protected. In addition, the activPAL data is fully de-identified. Once downloaded from the activPAL device, data will be immediately moved to the KPWHRI shared drive and deleted from personal computers.We have completed a Tech Risk Review which

deemed this activity from home, using Secure File Transfer from a home computer to a KPWHRI shared drive is considered low risk.

c. Does this study involve the disclosure of PHI to a collaborator?
If any data will be sent outside of this site, list each recipient (may list by role or category if the information is the same for several different entities). For each recipient, describe:
- What will be sent.
- Whether the information will be fully identifiable (PHI, if health information), a Limited Data Set, de-identified, or aggregate.
- How the data/materials will be transferred securely (for instance, Secure File Transfer).

Not applicable

7. Provisions to Monitor Data to Ensure the Safety of Subjects

This is required when research involves more than Minimal Risk to subjects.

The plan might include establishing a data monitoring committee and a plan for reporting data monitoring committee findings to the IRB and the sponsor.
Describe:
- Who will monitor the study data for safety?
- The plan to periodically evaluate the data collected regarding both harms and benefits to determine whether subjects remain safe.
- What data are reviewed, including safety data, untoward events, and efficacy data.
- How the safety information will be collected (e.g., with case report forms, at study visits, by telephone calls with participants).
- The frequency of data collection, including when safety data collection starts.
- The frequency or periodicity of review of cumulative data.
- Criteria for taking action on monitoring findings (for instance, stopping rules, immediate suspension, reporting, protocol changes, changes to monitoring frequency or plan).
- For studies monitored by a DSMB/C, describe the committee membership and structure, meeting format, and quorum requirements. Upload the board/committee charter, if one exists. (This bullet is not applicable)

Dr. Rosenberg, the PI, will be responsible for notifying the IRB of all adverse events (AEs). All participants learned to be deceased during the course of the study, regardless of the cause of death, will be reported to the IRB. The IRB will work with the PI to ensure additional agencies (e.g., NIH) are notified as required. The IRB will also determine if other immediate action is required (e.g., suspension of recruitment, protocol closure, etc.). Temporary or permanent suspension of an NIH-funded protocol will be reported to the NIH grant program director directly responsible for the grant.

Monitoring of Adverse Events by PI and Project Staff. Project staff (health coaches, research specialists, programmer, etc.) will be trained to identify potential AEs, document events on an adverse event form, and report any events immediately to the study PI and the project manager. Dr. Rosenberg will ensure that all AEs are appropriately handled and reported to the IRB and NIH authorities, as appropriate. The project manager will conduct routine protocol compliance

checks to ensure that safety procedures, such as ensuring participant confidentiality and maintaining approved standards for data transport, are strictly adhered to. In the case that the MD on call for the team (either Dr. Arterburn or Dr. Green) and /or the DSM Dr. James Ralston, determines that a participants lab values are out of range to an extent that they believe the participants provider should be notified, s/he may contact the provider (see the DSMP protocol) explaining the results of the test (example: high blood pressure). In order to do this, we need to collect the participant's provider name and have added this to the Contact Sheet.

Note: The study physician may determine what method of contacting the provider is most appropriate given the needs of the individual participant.

For the purposes of any Epic messages, only Drs Green and Nelson have Epic access and the only ones who would use this means of contacting a participant's provider.

<u>HIPAA Compliance</u>. All participants will be recruited from KPWA. As such, their participation is subject to the Health Insurance Portability and Accountability Act of 1996 (HIPAA) privacy and security standards. All KPWHRI staff have completed required HIPAA training, and all research activities will be conducted in compliance with the HIPAA standards.

<u>Monitoring Data Quality and Integrity</u>. Several procedures will be used to maintain data integrity. All databases will be stored in a centralized location on a departmental server at KPWHRI. Data will be backed up daily, and access to any participant information will be password protected and limited to persons working on the project. Project staff will only have access to specific data required for their project tasks. Identifying information will be stored separately from the assessment data. Data will be audited on an ongoing basis by the Project Manager to ensure confidentiality safeguards are being upheld and data integrity is being maintained. Data entry systems will be set up to allow field checks, range checks for continuous variables, valid value checks for categorical variables, and checks for logical consistency of responses. Queries and data reports will be generated on a routine basis to monitor data quality. REDCap data integrity safeguard: we are using the internal version of REDcap which is behind the KPWA firewall.

<u>Data Safety Monitoring Panel</u>. Dr. Rosenberg will monitor the study in compliance with IRB-approved procedures. A Data Safety Monitoring Panel, composed of two KPWHRI investigators who are not associated with the project, will review all adverse events and make final determinations as to whether any AEs are study related. On a quarterly basis during the course of the trial, the panel will evaluate the adverse event data to protect the safety of the study participants. It will make specific recommendations to the PI and study team regarding modifications to the intervention based on any observed adverse effects of the treatment under study.

8. Risks and Benefits

a. Risks to Subjects

List the reasonably foreseeable risks, discomforts, hazards, or inconveniences to the subjects related the subjects' participation in the research. Describe the probability, magnitude, duration, and reversibility of the risks. Consider physical, psychological, social, legal, and economic risks.

- If applicable, indicate which procedures may have risks to the subjects that are currently unforeseeable.
- If applicable, indicate which procedures may have risks to an embryo or fetus should the subject be or become pregnant.
- If applicable, describe risks to others who are not subjects and risks to Kaiser Permanente

The primary risks of participation include a breach of confidentiality; emotional upset or embarrassment due to participation in the research intervention, in-person measurements, and surveys; and potential adverse events related to participation in the intervention. People may feel uncomfortable wearing small devices that capture movement. The waterproof medical dressing used to secure the activPAL to the top of the thigh could result in skin irritation, though this outcome has rarely been reported in previous studies using activPAL and we use a medical grade adhesive. However, if this occurs, participants can discontinue use, try their other leg, or try a different adhesion method. Participants may feel uncomfortable when having their height and weight measured, performing brief physical function tasks, or completing a survey which asks about psychosocial factors, well-being, and quality of life. Persons who choose to increase their routine physical activity levels could experience physical-activity-related injuries such as pulled muscles or sprains. Participants may experience discomfort and/or local bruising at the site of the capillary finger stick blood draw.

For those participants with high or low blood pressure, the potential improvement in physical health is greater than potential adverse effects from increasing standing time. The goal of the i-STAND intervention is to increase breaks from sitting time. Study physicians do not think these, or any participants, are likely to experience harms greater than those encountered in normal life because standing up is a typical daily activity. Any unanticipated events that do occur will be documented in the AE form. At best, participants may be at increased risk for muscle stiffness/soreness or falls from standing/moving more, but longer term their physical health may benefit from reduced sedentary time.

In the event that a participant presents with any of these symptoms while waiting to be seen for their in-person measurement visit (e.g.: in the waiting room), the RS will follow the "Physical Health Concern Protocol" which entails contacting the KP Consulting Nurse Service, and follow directions from that nurse, and possibly rescheduling the measurement visit for another time.

The likelihood of breach of confidentiality is minimal, as outcome-oriented study data are identified only by study ID and files are password protected. Hard copies, audio files, and tracking data are retained in secured locations accessible only to project staff with a specific need for access. All KPWHRI staff receive training in the protection of confidentiality of research participants. Participants will be informed of all anticipated risks in the informed consent and provided with verbal and written safety information. Per the consent, if a participants lab values are out of range to an extent that the MD on call (Dr. Green or Dr. Arterburn) believes the participants provider should be notified, s/he will send a letter (included in the DSMP protocol) to the provider explaining the results of the test (example: high blood pressure).

b. Potential Benefits to Subjects

Describe the potential benefits that individual subjects may experience from taking part in the research. Include as may be useful for the IRB's consideration, the probability, magnitude, and duration of the potential benefits. Indicate if there is no direct benefit. You may include benefits to society or others.

All participants who consent to participate may receive some satisfaction or indirect benefit from contributing to this research. Additional benefits include advice and guidance on ways to reduce sedentary behavior and improve health. People who make lifestyle changes may experience other positive gains such as weight loss, reduced disease risk, improved mental health, and better management of existing chronic conditions. It is possible they may not experience any benefit at all.

9. Economic Burden to Subjects

Describe any costs that subjects may be responsible for because of participation in the research study (for example, co-pays; paying for treatment, therapies, or other interventions, or the delivery of these) and how you will inform participants of these costs prior to their enrollment in this study.

Not applicable. Participants will have parking validated and/or bus/light-rail tokens provided.

10. Compensation to Participants

Describe any compensation provided to participants, for example, for time inconvenience, discomfort, travel, or in the event of research related injury. If applicable, describe how you will inform participants of this prior to their enrollment in the study, including if payment will be prorated if the subject withdraws early from the study.

NOTE: payment may not be withheld as an incentive for participants to complete the study.

Participants will receive $30 incentives for completing measurements after each of the 4 measurement visits and a $30 bonus for completing all visits ($150 total). Finally, participants in both intervention arms will be offered the written materials for the intervention they did not receive at the end of the study. i-STAND participants will be asked to return both the wrist-worn activity band and the standing desk.

2022 BUDGET CUT UPDATE: As of March 31, 2022, there will no longer be a 12-month visit, bringing the new total amount given to participants to $120. We will continue to give a $30 bonus for completing the other timepoints (Baseline, 3 and 6 months).

11. Resources Available

Describe any special resources or expertise required to conduct the study.

Not applicable

12. Prior Approvals

Describe any approvals that will be obtained prior to commencing the research. (e.g., school, external site. funding agency, laboratory, radiation safety, or biosafety approval.)

Not applicable.

13. Drugs or Devices

NOTE: see the ICH-GCP guidance for a summary of investigator and sponsor responsibilities in clinical trials.

a. Drug Studies

If the research involves drugs and is investigator-initiated, indicate whether there is any possibility that the results will be reported to FDA (e.g. as part of a new drug application [NDA]).

- If the drug is investigational (has an IND), confirm that you will comply with all applicable FDA requirements for investigators.
- Confirm that you will follow applicable KP pharmacy policies and procedures.
- Describe your plan for drug storage, handling, and accountability, including distribution, return, and destruction of the drug(s).

Not applicable

b. Device Studies:

If this is a device study and you think the device is Non-Significant Risk, include justification here or upload it as a separate document along with any available device information (instructions for use, etc.).

If the research involves devices and is investigator-initiated, indicate whether there is any possibility that the results will be reported to FDA (e.g. as part of a premarket approval application [PMA]).

- If the device has an IDE or a claim of abbreviated IDE (Non-Significant Risk device), confirm that you will comply with all applicable FDA requirements for investigators.
- Describe the device, the manufacturing process, and the device labeling, including safety instructions or warnings. If available, this may be addressed in separately uploaded device information (such as instructions for use).
- Describe device storage, handling, and accountability, including how access to the device will be limited to appropriate personnel and how you will ensure the device will be used only for appropriate study subjects.

Not applicable

14. Multi-Site Research

a. If this is a multi-site study and you are the lead investigator or this site will be the coordinating center for any activity, describe the processes to ensure communication among sites, such as:
   - All sites have the most current version of the protocol, consent document, and HIPAA authorization.

- All required approvals have been obtained at each site (including approval by the site's IRB of record).
- All modifications have been communicated to sites, and approved (including approval by the site's IRB of record) before the modification is implemented.
- All engaged participating sites will safeguard data as required by local information security policies.
- All local site investigators conduct the study appropriately.

b. Describe the method for communicating to engaged participating sites the following:
- Problems.
- Interim results.
- The closure of a study.

c. Describe any special resources or expertise required to conduct the study.

Not applicable

15. Community-Based Participatory Research

Describe involvement of the community in the design and conduct of the research.

Describe your plan for ensuring that community research partners are appropriately trained in human subjects' protection.

NOTE: "Community-based Participatory Research" is a collaborative approach to research that equitably involves all partners in the research process and recognizes the unique strengths that each brings. Community-based Participatory Research begins with a research topic of importance to the community, has the aim of combining knowledge with action and achieving social change to improve health outcomes and eliminate health disparities.

Not applicable

1. Rosenberg DE, Gell NM, Jones SM, et al. The Feasibility of Reducing Sitting Time in Overweight and Obese Older Adults. *Health Educ Behav.* 2015;42(5):669-676.
2. Hamilton MT, Hamilton DG, Zderic TW. Sedentary behavior as a mediator of type 2 diabetes. *Med Sport Sci.* 2014;60:11-26.
3. Healy GN, Winkler EA, Owen N, Anuradha S, Dunstan DW. Replacing sitting time with standing or stepping: associations with cardio-metabolic risk biomarkers. *Eur Heart J.* 2015.
4. Flegal KM, Carroll MD, Ogden CL, Curtin LR. Prevalence and trends in obesity among US adults, 1999-2008. *JAMA.* 2010;303(3):235-241.
5. Flegal KM, Carroll MD, Kit BK, Ogden CL. Prevalence of obesity and trends in the distribution of body mass index among US adults, 1999-2010. *JAMA.* 2012;307(5):491-497.
6. Nicklas BJ, Gaukstern JE, Legault C, Leng I, Rejeski WJ. Intervening on spontaneous physical activity to prevent weight regain in older adults: Design of a randomized, clinical trial. *Contemp Clin Trials.* 2011;33(2):450-455.

7. Physical Activity Guidelines Committee. *Physical activity guidelines advisory committee report, 2008.* Washington, D.C.: U.S. Department of Health and Human Services;2008.
8. Troiano RP, Berrigan D, Dodd KW, Masse LC, Tilert T, McDowell M. Physical activity in the United States measured by accelerometer. *Med Sci Sports Exerc.* 2008;40(1):181-188.
9. Federal Interagency Forum on Aging-Related Statistics. *Older Americans 2010: key indicators of well-being.* Washington, D.C.: Federal Interagency Forum on Aging-Related Statistics;2010.
10. Brownson RC, Boehmer TK, Luke DA. Declining rates of physical activity in the United States: what are the contributors? *Annu Rev Public Health.* 2005;26:421-443.
11. Tudor-Locke C, Brashear MM, Johnson WD, Katzmarzyk PT. Accelerometer profiles of physical activity and inactivity in normal weight, overweight, and obese U.S. men and women. *Int J Behav Nutr Phys Act.* 2010;7:60.
12. Baert V, Gorus E, Mets T, Geerts C, Bautmans I. Motivators and barriers for physical activity in the oldest old: a systematic review. *Ageing Res Rev.* 2011;10(4):464-474.
13. Hootman JM, Driban JB, Sitler MR, Harris KP, Cattano NM. Reliability and validity of three quality rating instruments for systematic reviews of observational studies. *Research synthesis methods.* 2011;2(2):110-118.
14. Brawley LR, Rejeski WJ, King AC. Promoting physical activity for older adults: the challenges for changing behavior. *Am J Prev Med.* 2003;25(3 Suppl 2):172-183.
15. Biswas A, Oh PI, Faulkner GE, et al. Sedentary Time and Its Association With Risk for Disease Incidence, Mortality, and Hospitalization in Adults: A Systematic Review and Meta-analysis. *Ann Intern Med.* 2015;162(2):123-132.
16. Neuhaus M, Eakin EG, Straker L, et al. Reducing occupational sedentary time: a systematic review and meta-analysis of evidence on activity-permissive workstations. *Obes Rev.* 2014;15(10):822-838.
17. Mainsbridge CP, Cooley PD, Fraser SP, Pedersen SJ. The effect of an e-health intervention designed to reduce prolonged occupational sitting on mean arterial pressure. *J Occup Environ Med.* 2014;56(11):1189-1194.
18. Gorman E, Ashe MC, Dunstan DW, et al. Does an 'activity-permissive' workplace change office workers' sitting and activity time? *PLoS One.* 2013;8(10):e76723.
19. Greenwood-Hickman MA, Renz A, Rosenberg D. Motivators and barriers to reducing sedentary behavior among overweight and obese older adults. *Gerontologist.* 2016;56(4):660-668.
20. Matson TE, Renz AD, Takemoto ML, McClure JB, Rosenberg DE. Acceptability of a sitting reduction intervention for older adults with obesity. *BMC Public Health.* 2018;18(1):706.
21. Kerr J, Takemoto M, Bolling K, et al. Two-Arm Randomized Pilot Intervention Trial to Decrease Sitting Time and Increase Sit-To-Stand Transitions in Working and Non-Working Older Adults. *PLoS One.* 2016;11(1):e0145427.
22. Katon W, Lin EH, Von Korff M, et al. Integrating depression and chronic disease care among patients with diabetes and/or coronary heart disease: the design of the TEAMcare study. *Contemp Clin Trials.* 2010;31(4):312-322.
23. Vitiello MV, McCurry SM, Shortreed SM, et al. Cognitive-behavioral treatment for comorbid insomnia and osteoarthritis pain in primary care: the lifestyles randomized controlled trial. *J Am Geriatr Soc.* 2013;61(6):947-956.
24. McClure JB, Catz SL, Ludman EJ, Richards J, Riggs K, Grothaus L. Feasibility and acceptability of a multiple risk factor intervention: the Step Up randomized pilot trial. *BMC Public Health.* 2011;11:167.
25. Von Korff M, Balderson BH, Saunders K, et al. A trial of an activating intervention for chronic back pain in primary care and physical therapy settings. *Pain.* 2005;113(3):323-330.

26. Sternfeld B, LaCroix A, Caan BJ, et al. Design and methods of a multi-site, multi-behavioral treatment trial for menopausal symptoms: the MsFLASH experience. *Contemp Clin Trials.* 2013;35(1):25-34.
27. Guthrie KA, LaCroix AZ, Ensrud KE, et al. Pooled Analysis of Six Pharmacologic and Nonpharmacologic Interventions for Vasomotor Symptoms. *Obstetrics and gynecology.* 2015;126(2):413-422.
28. Arterburn D, Flum DR, Westbrook EO, et al. A population-based, shared decision-making approach to recruit for a randomized trial of bariatric surgery versus lifestyle for type 2 diabetes. *Surgery for obesity and related diseases : official journal of the American Society for Bariatric Surgery.* 2013;9(6):837-844.
29. Resnicow K, DiIorio C, Soet JE, Ernst D, Borrelli B, Hecht J. Motivational interviewing in health promotion: it sounds like something is changing. *Health Psychol.* 2002;21(5):444-451.
30. Miller WR, Rollnick S. *Motivational interviewing, second edition: preparing people for change.* 2nd ed. New York: The Guilford Press; 2002.
31. Valsamakis G, Chetty R, Anwar A, Banerjee AK, Kumar S. Association of simple anthropometric measures of obesity with visceral fat and the metabolic syndrome in male Caucasian and Indo-Asian subjects. *Diabet Med.* 2004;21(12):1339-1345.
32. Gardiner PA. Understanding and influencing sedentary behaviour in older adults [dissertation]. Vol Doctorate of Philosophy. Brisbane, Australia: University of Queeensland; 2011.
33. Salmon J, Hume C, Ball K, Booth M, Crawford D. Individual, social and home environment determinants of change in children's television viewing: the Switch-Play intervention. *J Sci Med Sport.* 2006;9(5):378-387.
34. Norman GJ, Sallis JF, Gaskins R. Comparability and reliability of paper- and computer-based measures of psychosocial constructs for adolescent physical activity and sedentary behaviors. *Res Q Exerc Sport.* 2005;76(3):315-323.
35. Norman G, Vaughn AA, Roesch S, Sallis JF, Calfas K, Patrick K. Development of decisional balance and self-efficacy measures for adolescent sedentary behaviors. *Psychol Health.* 2004;19:561-575.
36. Verplanken B, Orbell S. Reflections on past behavior: A self-report index of habit strength. *J Appl Soc Psychol.* 2003;33:1313-1330.
37. Guralnik JM, Ferrucci L, Simonsick EM, Salive ME, Wallace RB. Lower-extremity function in persons over the age of 70 years as a predictor of subsequent disability. *N Engl J Med.* 1995;332(9):556-561.
38. Guralnik JM, Simonsick EM, Ferrucci L, et al. A short physical performance battery assessing lower extremity function: association with self-reported disability and prediction of mortality and nursing home admission. *J Gerontol.* 1994;49(2):M85-M94.
39. Rosenberg DE, Bellettiere J, Gardiner PA, Villarreal VN, Crist K, Kerr J. Independent associations between sedentary behaviors and mental, cognitive, physical and functional health among older adults in retirement communities. *J Gerontol A Biol Sci Med Sci.* 2015;71(1):78-83.
40. Rosenberg DE, Norman GJ, Wagner N, Patrick K, Calfas KJ, Sallis JF. Reliability and validity of the Sedentary Behavior Questionnaire (SBQ) for adults. *J Phys Act Health.* 2010;7(6):697-705.
41. Kroenke K, Strine TW, Spitzer RL, Williams JB, Berry JT, Mokdad AH. The PHQ-8 as a measure of current depression in the general population. *J Affect Disord.* 2009;114(1-3):163-173.
42. Kozey-Keadle S, Libertine A, Lyden K, Staudenmayer J, Freedson PS. Validation of wearable monitors for assessing sedentary behavior. *Med Sci Sports Exerc.* 2011;43(8):1561-1567.
43. Hart TL, Ainsworth BE, Tudor-Locke C. Objective and subjective measures of sedentary behavior and physical activity. *Med Sci Sports Exerc.* 2011;43(3):449-456.
44. Grant PM, Dall PM, Mitchell SL, Granat MH. Activity-monitor accuracy in measuring step number and cadence in community-dwelling older adults. *J Aging Phys Act.* 2008;16(2):201-214.

45. Kozey-Keadle S, Libertine A, Staudenmayer J, Freedson P. The feasibility of reducing and measuring sedentary time among overweight, non-exercising office workers. *J Obes.* 2012;2012:282303.
46. Larkin L, Nordgren B, Purtill H, Brand C, Fraser A, Kennedy N. Criterion Validity of the activPAL Activity Monitor for Sedentary and Physical Activity Patterns in People Who Have Rheumatoid Arthritis. *Phys Ther.* 2015;96(7):1093-1101.
47. Lyden K, Kozey Keadle SL, Staudenmayer JW, Freedson PS. Validity of two wearable monitors to estimate breaks from sedentary time. *Med Sci Sports Exerc.* 2012;44(11):2243-2252.
48. Judice PB, Santos DA, Hamilton MT, Sardinha LB, Silva AM. Validity of GT3X and Actiheart to estimate sedentary time and breaks using ActivPAL as the reference in free-living conditions. *Gait Posture.* 2015;41(4):917-922.
49. Rejeski WJ, Brubaker PH, Goff DC, Jr., et al. Translating weight loss and physical activity programs into the community to preserve mobility in older, obese adults in poor cardiovascular health. *Arch Intern Med.* 2011;171(10):880-886.
50. Sink KM, Espeland MA, Castro CM, et al. Effect of a 24-Month Physical Activity Intervention vs Health Education on Cognitive Outcomes in Sedentary Older Adults: The LIFE Randomized Trial. *JAMA.* 2015;314(8):781-790.
51. Liang KY, Zeger S. Longitudinal data analysis using generazlied linear models. *Biometrika.* 1986;73(1):13-22.
52. Wang M, Fitzmaurice GM. A simple imputation method for longitudinal studies with non-ignorable non-responses. *Biom J.* 2006;48(2):302-318.
53. Baron RM, Kenny DA. The moderator-mediator variable distinction in social psychological research: conceptual, strategic, and statistical considerations. *J Pers Soc Psychol.* 1986;51(6):1173-1182.
54. Preacher KJ, Hayes AF. Asymptotic and resampling strategies for assessing and comparing indirect effects in multiple mediator models. *Behav Res Methods.* 2008;40(3):879-891.
55. Figueira FR, Umpierre D, Cureau FV, et al. Association between physical activity advice only or structured exercise training with blood pressure levels in patients with type 2 diabetes: a systematic review and meta-analysis. *Sports Med.* 2014;44(11):1557-1572.
56. Semlitsch T, Jeitler K, Hemkens LG, et al. Increasing physical activity for the treatment of hypertension: a systematic review and meta-analysis. *Sports Med.* 2013;43(10):1009-1023.
57. Murtagh EM, Nichols L, Mohammed MA, Holder R, Nevill AM, Murphy MH. The effect of walking on risk factors for cardiovascular disease: an updated systematic review and meta-analysis of randomised control trials. *Prev Med.* 2015;72:34-43.
58. Lewington S, Clarke R, Qizilbash N, Peto R, Collins R, Prospective Studies C. Age-specific relevance of usual blood pressure to vascular mortality: a meta-analysis of individual data for one million adults in 61 prospective studies. *Lancet.* 2002;360(9349):1903-1913.
