# Supplementary material for: Sitting Time Reduction and Blood Pressure in Older Adults: A Randomized Clinical Trial
Source: JAMA Netw Open. 2024 Mar 27;7(3):e243234. doi: 10.1001/jamanetworkopen.2024.3234 (PMC10973891; doi:10.1001/jamanetworkopen.2024.3234)
Supplement: Supplement 2. — eTable 1. Adherence to Health Coaching Sessions and Fidelity to the Interventions eTable 2. Coprimary Outcome Secondary Analysis at 3 and 6 Months by Intervention Group and Randomization Pre or Post COVID-19 Pandemic eTable 3. Coprimary Outcomes Results by Intervention Group at 3 and 6 Months by Whether All Measurements Were Completed Before or After the COVID-19 Pandemic Onset eTable 4. Moderator Analyses at 6 Months to Assess Differential Intervention Effects eFigure. Waterfall Plot of Changes From Baseline to 6 Months in Mean Daily Minutes of Sitting Time [file jamanetwopen-e243234-s002.pdf]

Supplementary Online Content

Rosenberg DE, Zhu W, Greenwood-Hickman MA, et al. Sitting time reduction and blood pressure in older adults: a randomized clinical trial. *JAMA Netw Open*. 2024;7(3):e243234. doi:10.1001/jamanetworkopen.2024.3234

eTable 1. Adherence to Health Coaching Sessions and Fidelity to the Interventions

eTable 2. Coprimary Outcome Secondary Analysis at 3 and 6 Months by Intervention Group and Randomization Pre or Post COVID-19 Pandemic[a]

eTable 3. Coprimary Outcomes Results by Intervention Group at 3 and 6 Months by Whether All Measurements Were Completed Before or After the COVID-19 Pandemic Onset

eTable 4. Moderator Analyses at 6 Months to Assess Differential Intervention Effects

eFigure. Waterfall Plot of Changes From Baseline to 6 Months in Mean Daily Minutes of Sitting Time

This supplementary material has been provided by the authors to give readers additional information about their work.

© 2024 Rosenberg DE et al. *JAMA Network Open.*

eTable 1. Adherence to Health Coaching Sessions and Fidelity to the Interventions

	Overall %[a]	I-STAND Intervention %[a]	Healthy Living Control %[a]
**Total sample**			
Total sessions, n	2432	1245	1187
Mean number of sessions, mean (SD)		8.9 (2.0)	8.3 (2.4)
Adherent to 8+ sessions*, n (%)		121 (86.4)	110 (76.9)
**Fidelity coding sample**			
Sessions Reviewed, n	221	136	85
Unique Participants, n	96	58	38
Overall Session Rating[b], mean (SD)	2.9 (0.2)	2.9 (0.3)	3.0 (0.2)

*The interventions included 10 health coaching sessions in both arms

[a] Percentage of sessions reviewed that met the indicated criteria.

[b] Session-rating scale: 0 = did not follow content; 1 = addressed some content but skipped key concepts; 2 = mainly followed content with minor changes; 3 = followed content as directed in health coach training and materials

© 2024 Rosenberg DE et al. *JAMA Network Open.*

eTable 2. Coprimary Outcome Secondary Analysis at 3 and 6 Months by Intervention Group and Randomization Pre or Post COVID-19 Pandemic[a]

	PRE COVID		POST COVID	
**Measure**	Difference in Adjusted Mean Changes[b] (95% CI)	P-Value	Difference in Adjusted Mean Changes[b] (95% CI)	P-Value
Sitting Time (N = 246)				
3 months	-50.46 (-81.75,-19.17)	0.002	-21.79 (-42.55,-1.02)	0.04
6 months*	-15.86 (-55.29,23.56)	0.43	-40.25 (-65.39,-15.11)	0.002
SBP (N = 250)				
6 months**	-3.41 (-9.47,2.66)	0.271	-3.75 (-7.27,-0.23)	0.04
DBP (N = 250)				
6 months**	1.24 (-2.04,4.53)	0.458	-0.40 (-2.66,1.85)	0.73

[a] 100 randomized before covid and 183 after covid. In the regression model, due to the missing of covariate and outcomes, a total of 246 participants (sitting time) and 250 participants (blood pressure) data were used.

[b] Difference in adjusted mean change: Difference between adjusted mean change in I-STAND intervention minus adjusted mean change in attention control at the stated timepoint for those randomized pre COVID-19 pandemic or those randomized in March 2020. Results were calculated fitting a linear regression model with generalized estimating equations on the outcome change from baseline including timepoints 3 and 6 months in the same model with indicators for 6-month timepoint, I-STAND intervention, post-COVID randomization, and interactions between indicators adjusting for baseline outcome, baseline outcome and post-COVID randomization interaction, county, age, gender, race, body mass index, arthritis, physical function, diabetes, hypertension, retirement status and number of hypertensive medication classes. To obtain the adjustment mean change, we assumed the mean baseline covariate response for all randomized. For SBP and DBP only 6-month outcomes were included in the analyses. CI, confidence interval; SBP, systolic blood pressure; DBP, diastolic blood pressure

*6 months is the primary outcome timepoint of the trial

** Blood pressure outcomes pre COVID were only collected only at the 6-month timepoint

© 2024 Rosenberg DE et al. *JAMA Network Open.*

eTable 3. Coprimary Outcomes Results by Intervention Group at 3 and 6 Months by Whether All Measurements Were Completed Before or After the COVID-19 Pandemic Onset

Measure	Attention Control<br>Adjusted Mean Change[a] (95% CI)	I-STAND Intervention<br>Adjusted Mean Change[a] (95% CI)	Difference in Adjusted Mean Changes[a] (95% CI)	P-Value
Sitting Time				
PRE-COVID all measurements (N= 45)				
3 months	13.12 (-12.47,38.72)	-70.47 (-99.14,-41.80)	-83.60 (-124.36,-42.83)	<0.001
6 months*	9.14 (-24.95,43.23)	-48.11 (-88.21,-8.02)	-57.25 (-113.78,-0.73)	0.05
POST-COVID all measurements (N = 163)				
3 months	-9.02 (-21.51,3.47)	-31.69 (-47.42,-15.96)	-22.67 (-43.20,-2.14)	0.03
6 months*	-3.24 (-20.71,14.23)	-43.62 (-61.82,-25.42)	-40.38 (-65.24,-15.52)	0.001
SBP				
PRE-COVID all measurements (N = 47)				
6 months*	1.66 (-5.22, 8.53)	1.01 (-4.32, 6.35)	-0.64 (-10.58, 9.29)	0.90
POST-COVID all measurements (N = 166)				
3 months**	-5.16 (-7.81, -2.51)	-3.29 (-6.19, -0.39)	1.87 (-2.08, 5.82)	0.35
6 months*	-4.52 (-7.01, -2.02)	-7.70 (-10.19, -5.20)	-3.18 (-6.75, 0.39)	0.08
DBP				
PRE-COVID all measurements (N = 47)				
6 months*	2.06 (-1.86, 5.97)	1.48 (-1.10, 4.07)	-0.57 (-5.79, 4.65)	0.83
POST-COVID all measurements (N=166)				
3 months**	-2.80 (-4.21 ,-1.39)	-1.11 (-3.12, 0.90)	1.69 (-0.83, 4.21)	0.19
6 months*	-2.02 (-3.59, -0.45)	-2.02 (-3.70, -0.33)	0.00 (-2.34, 2.34)	1

[a] Adjusted mean change and difference: Calculated fitting a separate model for the subgroup with PRE-COVID all measurements and the subgroup with POST-COVID all measurements. Model fit was a linear regression model with generalized estimating equations on the outcome change from baseline, including timepoints 3 and 6 months in the same model with indicators for 6-month timepoint, I-STAND intervention, and interactions between these indictors adjusting for baseline outcome, county (for POST-COVID subgroup only), age, gender, race, body mass index, arthritis, physical function, diabetes, hypertension, retirement status and number of hypertensive medication classes. To obtain the adjustment mean change we assumed the mean baseline covariate response for all randomized. For SBP and DBP PRE-COVID models, only 6-month outcomes were included in analyses. CI, confidence interval; SBP, systolic blood pressure; DBP, diastolic blood pressure

* 6 months is the primary timepoint of the trial

** Blood pressure for participants randomized before the COVID-19 pandemic was measured at only the 6-month timepoint

© 2024 Rosenberg DE et al. *JAMA Network Open.*

eTable 4. Moderator Analyses at 6 Months to Assess Differential Intervention Effects

	SITTING TIME		SBP		DBP	
**Measure**	Difference in Adjusted Mean Changes[a] (95% CI)	P-value[b]	Difference in Adjusted Mean Changes[a] (95% CI)	P-value[b]	Difference in Adjusted Mean Changes[a] (95% CI)	P-value[b]
Fall in past 12 mo		0.34		0.9		0.24
Yes	-18.09 (-49.24,13.06)		-3.63 (-8.74,1.48)		-1.34 (-4.46,1.79)	
No	-38.07 (-65.38,-10.76)		-4.03 (-7.94,-0.12)		0.94 (-1.38,3.27)	
Hypertension		0.32		0.84		0.68
Yes	-19.95 (-48.46,8.57)		-3.30 (-7.95,1.34)		0.56 (-2.18,3.30)	
No	-42.56 (-74.74,-10.38)		-3.95 (-8.13,0.22)		-0.21 (-2.76,2.33)	
Antihypertensive meds		0.61		0.66		0.88
Yes	-34.32 (-58.75,-9.90)		-4.26 (-8.08,-0.45)		0.22 (-2.12,2.56)	
No	-22.17 (-61.80,17.47)		-2.73 (-8.42,2.96)		-0.11 (-3.49,3.27)	
Diabetes		0.62		0.78		0.38
Yes	-38.82 (-72.57,-5.07)		-2.88 (-9.00,3.23)		1.50 (-2.00,5.00)	
No	-27.80 (-54.10,-1.50)		-3.92 (-7.65,-0.20)		-0.35 (-2.56,1.87)	
Able to walk at normal pace		0.15		0.14		0.3
Yes	-21.86 (-46.11,2.39)		-2.36 (-5.80,1.07)		0.77 (-1.26,2.79)	
No	-68.08 (-122.30,-13.85)		-8.49 (-15.87,-1.10)		-2.10 (-7.06,2.86)	
Age		0.09		0.79		0.78
60-74	-20.64 (-46.17,4.90)		-3.86 (-7.45,-0.27)		0.06 (-1.97,2.09)	
75+	-70.29 (-116.98,-23.59)		-2.79 (-9.78,4.19)		0.85 (-4.21,5.91)	
Gender		0.33		0.08		0.003

© 2024 Rosenberg DE et al. *JAMA Network Open.*

Women	-37.61 (-63.96,-11.26)		-5.57 (-9.37,-1.77)		-1.76 (-4.06,0.55)	
Men	-16.44 (-49.72,16.84)		0.31 (-5.24,5.86)		4.12 (0.94,7.30)	
Retirement status		0.62		0.11		0.8
Retired	-35.91 (-62.25,-9.57)		-1.46 (-5.54,2.61)		-0.02 (-2.60,2.55)	
Working	-23.60 (-61.42,14.23)		-6.65 (-11.53,-1.77)		0.44 (-2.17,3.06)	
County location		0.24		0.18		0.03
King	-42.77 (-72.43,-13.12)		-1.68 (-6.06,2.70)		2.04 (-0.56,4.65)	
Outside King	-16.84 (-47.40,13.73)		-5.97 (-10.44,-1.51)		-2.06 (-4.76,0.63)	
Physical activity		0.47		0.98		0.84
<5000 steps/day	-37.87 (-66.52,-9.22)		-3.64 (-8.01,0.74)		0.29 (-2.40,2.98)	
≥5000 steps/day	-22.80 (-52.87,7.26)		-3.70 (-8.48,1.07)		0.67 (-1.95,3.29)	

Mo, months; CI, confidence interval; SBP, systolic blood pressure; DBP, diastolic blood pressure

[a] Difference in adjusted mean change: This is the difference between the adjusted mean change in I-STAND intervention minus the adjusted mean change in the attention control within a given moderator category. We fit a linear regression model with GEE on the outcome change from baseline including only 6 months outcome data with an indicator for the moderator, indicator for I-STAND intervention, and interactions between these indicators adjusting for baseline outcome, baseline outcome and Post-COVID randomization interaction, county, age, gender, race, BMI, arthritis, physical function, diabetes, hypertension, retirement status and number of hypertensive classes.

[b] Interaction p-value tests if the interaction effect between the moderator indicator and intervention indicator are statistically significant

© 2024 Rosenberg DE et al. *JAMA Network Open.*

Supplementary Figure 1
Waterfall plot of changes from baseline to 6−months in mean daily minutes of sitting time

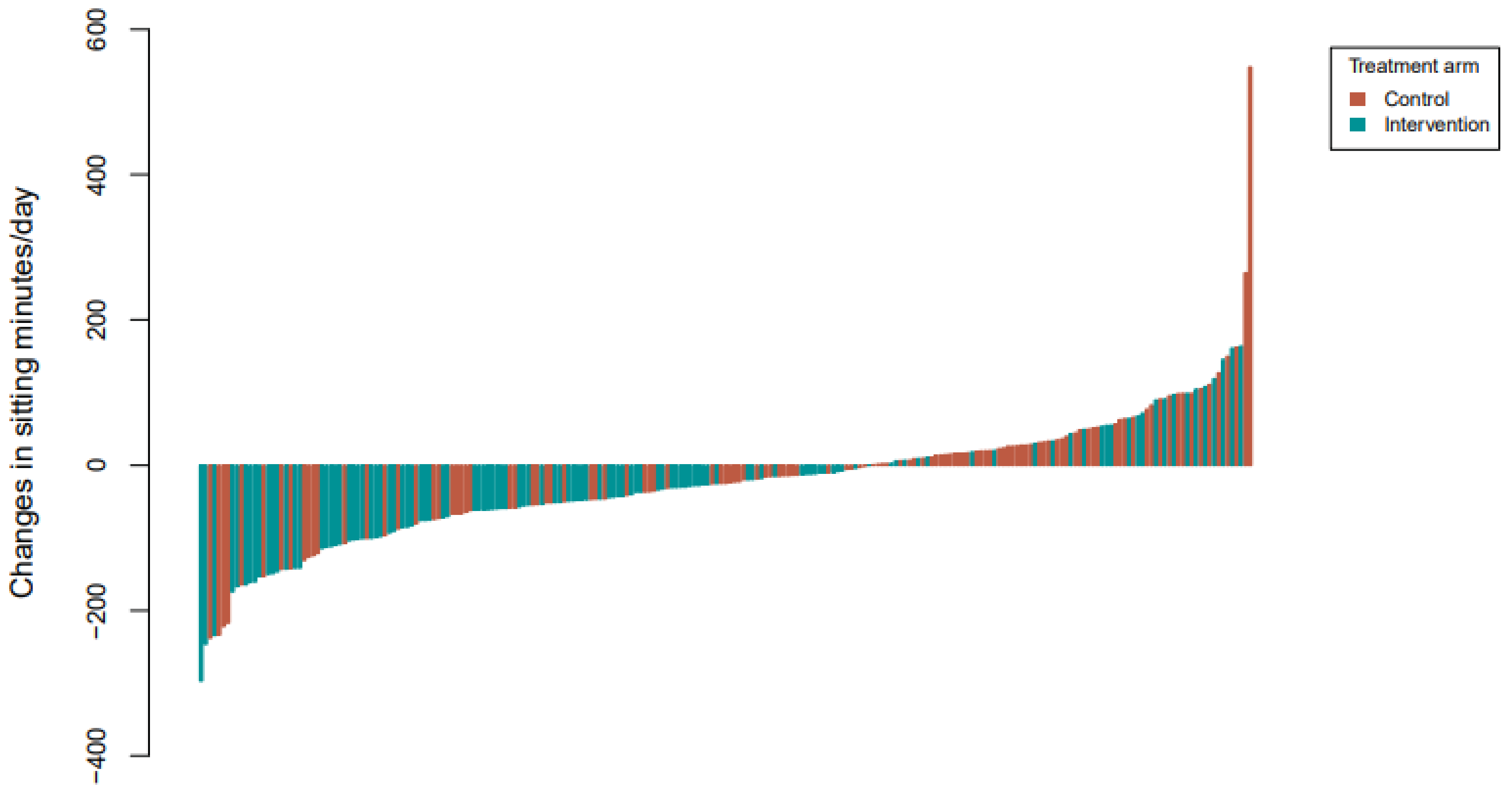

© 2024 Rosenberg DE et al. *JAMA Network Open.*
