# Supplementary material for: Sitting Time Reduction and Blood Pressure in Older Adults: A Randomized Clinical Trial
Source: JAMA Netw Open. 2024 Mar 27;7(3):e243234. doi: 10.1001/jamanetworkopen.2024.3234 (PMC10973891; doi:10.1001/jamanetworkopen.2024.3234)
Supplement: Supplement 3. — Data Sharing Statement [file jamanetwopen-e243234-s003.pdf]

Data Sharing Statement

Rosenberg. Sitting Time Reduction and Blood Pressure in Older Adults. *JAMA Netw Open*. Published March 27, 2024. doi:10.1001/jamanetworkopen.2024.3234

Data
Data available: No

Additional Information
Explanation for why data not available: There is a potential to share the data upon reasonable request to the corresponding author and after execution of needed data use agreements.
